# Over-expressed, N-terminally truncated BRAF is detected in the nucleus of cells with nuclear phosphorylated MEK and ERK

**DOI:** 10.1016/j.heliyon.2018.e01065

**Published:** 2018-12-20

**Authors:** Fiona Hey, Catherine Andreadi, Catherine Noble, Bipin Patel, Hong Jin, Tamihiro Kamata, Kees Straatman, Jinli Luo, Kathryn Balmanno, David T.W. Jones, V. Peter Collins, Simon J. Cook, Christopher J. Caunt, Catrin Pritchard

**Affiliations:** a Department of Molecular Cell Biology, University of Leicester, Lancaster Road, Leicester LE1 9HN, UK; b Leicester Cancer Research Centre, Clinical Sciences Building, University of Leicester, Leicester Royal Infirmary, Leicester LE2 7LX, UK; c Core Biotechnology Services, University of Leicester, Lancaster Road, Leicester LE1 9HN, UK; d Signalling Laboratory, The Babraham Institute, Babraham Research Campus, Cambridge CB22 3AT, UK; e Department of Pathology, Division of Molecular Histopathology, University of Cambridge, Cambridge CB2 0QQ, UK; f Department of Biology and Biochemistry, University of Bath, Claverton Down, Bath BA2 7AY, UK

**Keywords:** Biochemistry, Cell biology

## Abstract

BRAF is a cytoplasmic protein kinase, which activates the MEK-ERK signalling pathway. Deregulation of the pathway is associated with the presence of *BRAF* mutations in human cancer, the most common being ^*V600E*^*BRAF*, although structural rearrangements, which remove N-terminal regulatory sequences, have also been reported. RAF-MEK-ERK signalling is normally thought to occur in the cytoplasm of the cell. However, in an investigation of BRAF localisation using fluorescence microscopy combined with subcellular fractionation of Green Fluorescent Protein (GFP)-tagged proteins expressed in NIH3T3 cells, surprisingly, we detected N-terminally truncated BRAF (ΔBRAF) in both nuclear and cytoplasmic compartments. In contrast, ΔCRAF and full-length, wild-type BRAF (^WT^BRAF) were detected at lower levels in the nucleus while full-length ^V600E^BRAF was virtually excluded from this compartment. Similar results were obtained using ΔBRAF tagged with the hormone-binding domain of the oestrogen receptor (hbER) and with the KIAA1549-ΔBRAF translocation mutant found in human pilocytic astrocytomas. Here we show that GFP-ΔBRAF nuclear translocation does not involve a canonical Nuclear Localisation Signal (NLS), but is suppressed by N-terminal sequences. Nuclear GFP-ΔBRAF retains MEK/ERK activating potential and is associated with the accumulation of phosphorylated MEK and ERK in the nucleus. In contrast, full-length GFP-^WT^BRAF and GFP-^V600E^BRAF are associated with the accumulation of phosphorylated ERK but not phosphorylated MEK in the nucleus. These data have implications for cancers bearing single nucleotide variants or N-terminal deleted structural variants of *BRAF*.

## 1 Introduction

There are three members of the mammalian RAF protein kinase family: ARAF, BRAF and CRAF. Oncogenic forms of the *RAFs*, encoding only their C-terminal kinase domains, were originally identified as transforming genes in oncogenic retroviruses of mice and chickens [1]. RAF proteins share three conserved regions (CR) 1–3, with CR3 encoding the kinase domains in the C-termini. This region is the most homologous amongst the three RAFs, showing >75% identity [2], and its activity is suppressed by the N-terminus [3].

RAF protein kinases play a key role in intracellular signalling by linking RAS activity with the Mitogen-activated protein kinase kinase (MEK)-Extracellular signal-Regulated Kinase (ERK) signalling pathway. Deregulation of the pathway is associated with numerous pathologies [4], most notably cancer, and *RAF* mutations are frequently found in cancer samples. In mice and chickens, as mentioned, this is evident in the form of N-terminal truncations, which generate oncoproteins with transforming activity. In human cancers, mutations in *BRAF* are more frequent than mutations in either *ARAF* or *CRAF* [5], most commonly through the acquisition of nucleotide substitutions such as that encoding the ^V600E^BRAF mutant [6]. Although N-terminal truncations of RAF are rare in human cancers, chromosomal translocations that delete the N-terminal regulatory domain of BRAF have been reported in multiple cancer types [7, 8] as well as aberrant spliced BRAF variants that delete the RAS binding domain in cells resistant to the BRAF inhibitor vemurafenib [9]. In the vast majority of cases, *BRAF* mutations are gain-of-function that mediate oncogenic effects through elevated BRAF kinase activity [8, 10, 11].

In a well-accepted model of RAF-MEK-ERK signalling [12], RAF is held in the cytoplasm in an inactive conformation bound to 14-3-3 through phosphorylated serine residues at S729 and S365. Following stimulation of cell surface receptors and the subsequent conversion of RAS to its GTP-bound active form, 14-3-3 binding to RAF is disrupted, RAS binds to the N-terminal domain of RAF, RAF dimerises and is translocated to the plasma membrane where it becomes active. RAF can then phosphorylate MEK1/2 at two serine residues in their activation loop and this active MEK phosphorylates threonine and tyrosine residues in the TEY motif of ERK1/2 to activate it. ERK is a pleiotropic kinase and can phosphorylate many substrates in nearly all cell compartments to elicit different biological effects [13, 14]. There is considerable evidence to show that cell cycle entry is dependent on the nuclear accumulation of active ERK, leading to phosphorylation of transcription factors and propagation of immediate early gene and protein expression [13, 15, 16].

The mechanism of ERK transport across the nuclear pore is complex, with evidence showing it occurs by energy-dependent and –independent mechanisms [17]. ERK lacks a canonical Nuclear Localisation Signal (NLS) and does not interact with importinαβ but relies on interaction with a range of proteins for appropriate localisation within the cell [18, 19, 20]. Energy-independent nuclear import of ERK is facilitated by interaction with nuclear pore proteins. Stimulus-dependent ERK nuclear import involves phosphorylation of ERK by MEK and disruption of the MEK-ERK association in the cytoplasm [21, 22] as well as abrogation of the interaction between ERK and other cytoplasmic anchors through ERK's D-domain [23]. A possible mechanism for ERK nuclear import may be through a Nuclear Translocation Signal (NTS) within an SPS motif in the ERK kinase insertion domain [24]. Phosphorylation of two serine residues in this motif has been suggested to allow interaction with importin7, release from interaction with nuclear pore proteins and subsequent nuclear entry [24]. MEK functions as a cytoplasmic anchor for ERK although it is also capable of entering the nucleus upon cellular stimulation and detachment from ERK [21, 24, 25]. However, MEK is exported from the nucleus much faster than ERK due to a nuclear export signal (NES), a leucine-rich sequence in its N-terminus [24, 25], that allows its rapid Crm1-dependent nuclear export.

Despite the overwhelming evidence supporting a cytoplasmic location of RAF proteins and their translocation to the plasma membrane upon activation [16, 26], there are reports of alternative locations within the cell. BRAF in particular has been detected in mitochondria [27], Golgi [28, 29], the mitotic spindle [30] and the nucleus [31, 32], and this compartmentalisation is associated with distinct biological outcomes in some circumstances [27, 30, 32]. For example, a portion of BRAF has been detected at spindle poles and kinetochores in mitotic HeLa cells and knockdown of BRAF using siRNA resulted in early exit of cells from mitosis, perturbation of Mps1 localisation and the formation of pleiotropic spindle abnormalities and misaligned chromosomes [30]. BRAF isoforms have also been detected in nuclear fractions of the rat forebrain and cerebellum [31] with a recent investigation identifying BRAF in the nucleus of skeletal muscle cells after activation, where it was found to interact with and phosphorylate PAX3 leading to enhancement of MET activity, a requirement for limb muscle precursor cell migration [32]. However, the relevance of these alternative locations for BRAF and their role in downstream MEK/ERK signaling and BRAF-driven oncogenesis has not been fully explored as yet.

In this study, we have used tagged, exogenously expressed RAF proteins in NIH3T3 cells combined with fluorescence microscopy and fractionation methods to evaluate BRAF compartmentalisation in more detail. Surprisingly, we detect the accumulation of N-terminally truncated forms of BRAF in the nucleus whereas full length, wild-type BRAF and ^V600E^BRAF are detected in the nucleus to a lower extent. Here, we correlate the compartmentalisation of these GFP-tagged forms of BRAF with the localisation of MEK and ERK in NIH3T3 cells.

## 2 Materials and methods

### 2.1 Vectors

To generate GFP-RAF expression vectors, cDNAs expressing wild-type or mutant versions of BRAF or CRAF were cloned into pEGFP-C1 vector (Clontech). GFP-ΔBRAF contains residues 449-804 of mouse BRAF, GFP-ΔCRAF contains residues 306-648 of human CRAF, GFP-FL-^WT^BRAF contains residues 1-766 of human BRAF and GFP-FL-^V600E^BRAF contains residues 1-766 of human BRAF with the V600E mutation. The human KIAA1549:BRAF and human ^WT^BRAF cDNAs cloned within the pcDNA3.1 expression vector have been reported previously [33]. Mutations within GFP-ΔBRAF or GFP-FL-^WT^BRAF were generated by performing site-directed mutagenesis using the GeneTailor^TM^ system (Thermo Fisher, 12397). Adenoviruses expressing human GFP-FL-^WT^BRAF or human GFP-ΔBRAF were generated by using the methods previously described [34].

### 2.2 Cell culture

NIH3T3 cells were grown in 4.5 g/L glucose DMEM containing 10% (v/v) Foetal Calf Serum and 1% (v/v) penicillin-streptomycin at 37 °C and 5% CO_2_. Mouse Embryonic Fibroblasts (MEFs) harbouring homozygous knockout mutations of *Araf* or *Craf* or wild-type controls were derived and cultured as reported previously [35]. MEFs expressing endogenous oncogenic ^G12D^KRAS or wild-type controls were derived and cultured as reported previously [36]. Cells were transfected using a Nucleofector under conditions recommended by the manufacturer (Amaxa Biosystems) as previously described [37]. NIH3T3 cells stably expressing ΔBRAF:ER or ΔCRAF:ER have been previously reported [38]. These cells were cultured as for NIH3T3 cells and were treated with 1 μM 4-hydroxytamoxifen (4-HT; Sigma, T-176) or 100% ethanol as carrier control for 24 or 48 hours before analysis. Cells were treated with 20 nM Leptomycin B (LMB; Cell Signaling, 9676) or carrier control (100% ethanol) for 3 hours before analysis in some experiments.

### 2.3 Fluorescence microscopy

Forty-eight hours after transfection with GFP vectors, cells were counterstained with 1 mg/ml 4′,6-diamidino-2-phenylindole (DAPI), processed and GFP/DAPI fluorescence was visualized by fluorescence microscopy as previously described [39]. For immunofluorescence of RAF:ER fusion proteins, cells were processed as previously described [40] and immunostained by indirect immunofluorescence with a rabbit polyclonal anti-ERα antibody (Santa Cruz SC-543) as the primary antibody and an AlexaFluor® 568-conjugated anti-rabbit IgG Fab as the secondary antibody before counterstaining with DAPI. The staining pattern in each cell was classified by user analysis into one of three categories: a) nuclear less than cytoplasmic (N < C), b) equally distributed (N=C), c) nuclear greater than cytoplasmic (N > C). Epifluorescence images were taken on an inverted Nikon TE300 microscope with Hamamatsu ORCA-ER digital camera and X-cite 120 fluorescence illumination system controlled by Improvision's Openlab software.

### 2.4 Protein analysis

For fractionation, cytoplasmic and nuclear fractions were prepared by using the NE-PER^TM^ kit (Thermo Fisher, 78833) following the manufacturer's instructions. Whole cell Triton X-100 soluble lysates were prepared as described [41]. For western blot analysis, the following antibodies were used: GFP (Abcam, ab6556), α-Tubulin (Sigma, T6074), PARP (Cell Signaling, 9542), BRAF (Santa Cruz, SC-5284), C-terminus of BRAF (Santa Cruz, SC-166), ER (Santa Cruz, SC-543), GAPDH (EMD Millipore, MAB374), Histone H1 (Santa Cruz, SC-8030), PP-MEK1/2 (Cell Signaling, 9154), MEK1/2 (Cell Signaling, 9122), PP-ERK1/2 (Cell Signaling, 9101) and ERK2 (Santa Cruz, SC-154). To confirm purity of fractions, western blots were analysed with antibodies for α-Tubulin or GAPDH as cytoplasmic markers or with PARP or Histone H1 as nuclear markers. For assessing the RAF kinase activity of GFP-ΔBRAF fractions or control GFP, nuclear/cytoplasmic fractions (GFP-ΔBRAF) or whole cell lysates (GFP) were subjected to immunoprecipitation using GFP–TRAP beads (Chromotek, ABIN509407) under their recommended conditions and the RAF kinase cascade assay was undertaken as previously described [26].

### 2.5 Quantitation of gel blots

Image J software was used to quantitate protein levels in western blots. After scanning and derivation of density measurements, the background on the blots was first subtracted from the pixel counts for each band and final values were divided by the values obtained for the respective loading controls. N:C proportions were determined by dividing nuclear values by total values for each sample. PP-MEK/total MEK and PP-ERK/ERK2 values were determined by dividing signals for PP-MEK or PP-ERK by signals for total MEK or ERK2 respectively.

### 2.6 Fluorescence recovery after photobleaching (FRAP)

NIH3T3 cells were transfected with vectors expressing either GFP-ΔBRAF or GFP alone, transferred to cover glass-bottomed Ibidi dishes and maintained in an environmental chamber with 5% CO_2_ at 37 °C. FRAP experiments were performed using a Leica TCS SP5 confocal laser scanning microscope attached to a Leica DMI 6000B inverted microscope using a 63 x oil objective. Five single optical sections were captured prior to bleaching a zoomed region of interest (ROI) containing the nucleus using 10 iterations and 100% laser power of the 488 nm argon laser. The recovery of the bleached nucleus was recorded by collecting 300 single optical sections with a time interval of 5s for 13 cells (GFP-ΔBRAF) or 11 cells (GFP). The recovery was corrected for background signal and loss in signal in a non-bleached area as a result of acquiring the images. The recovery was calculated as the corrected fluorescence intensity at a given time point divided by the corrected fluorescence intensity of the first frame before photobleaching. Mean mobile fractions and recovery half times were determined using Easy FRAP [42].

### 2.7 High content microscopy (HCM)

NIH3T3 cells were infected with adenoviruses expressing Ad-GFP-FL-^WT^BRAF or Ad-GFP-ΔBRAF for 4–6 hours and, 48 hours post-infection, they were seeded into 96 well black-wall imaging plates (Corning, 3904). Cells were treated with 20nM LMB for 3 hours before processing. Cells were immunostained with a primary antibody for PP-ERK1/2 (1:200 clone MAPK-YT, Sigma M9692) or PP-MEK (1:200, Cell Signaling, 9154) using AlexaFluor® 546-conjugated goat anti-mouse IgG (H + L) (1:200, Thermo Fisher, A-11003) or AlexaFluor® 546-conjugated goat anti-rabbit IgG (Fab) antibody (1:200, Thermo Fisher, A11018) and counterstained with DAPI as previously reported [34, 43]. Cells were imaged using an IN Cell Analyzer 2000 microscope (GE Healthcare) under 10x objective and excitation and emission filters of 360 and 460 nm for DAPI, 475 and 535 nm for GFP or 535 and 620 nm for Alexa 546. Automated image analysis algorithms were defined using IN Cell Developer software (GE Healthcare) to capture nuclear PP-ERK/PP-MEK and nuclear GFP fluorescence intensities (each within nuclear perimeters as defined by the DAPI stain). Fluorescence measures were reported in arbitrary fluorescence units (AFU). To test for a positive correlation between nuclear PP-ERK/PP-MEK and nuclear GFP, cells were sorted into bins according to the nuclear GFP stain (each bin spanning 50–100 AFU) and, for each bin, the mean GFP value was plotted against the mean PP-ERK/PP-MEK stain intensity in the binned cells.

### 2.8 Statistical analysis

Comparison between two groups was performed by the unpaired *t* test with Welch's correction using Prism software version 7. The data are presented as the mean value and the error bars indicate ±SD or ±SEM (as indicated). Significance is indicated as *** for *p* < 0.001, ** for *p* < 0.01, * for *p* < 0.05 and not significant (NS) for *p* values > 0.05. For HCM, correlation coefficients were determined and compared using R package cocor (cran.r-project.org/web/packages/cocor/index.html).

## 3 Results

### 3.1 Localisation of GFP-tagged RAF fusion proteins

To further understand the role of BRAF within the cell, we investigated its subcellular localisation. In initial experiments, immunofluorescence was performed using a well-utilised BRAF antibody that has previously been shown to detect BRAF protein species by Western blot analysis of wild-type MEFs that disappear in *Braf*^*−/−*^ MEFs [44]. Despite this apparent specificity, a weak immunofluorescence signal was detected in *Braf*^*−/−*^ cells with the same antibody, whereas no signal was seen with the secondary antibody alone (Fig. S1). These results show that immunofluorescence staining for BRAF is unreliable. We therefore resorted to using fluorescence microscopy of GFP-tagged, exogenously expressed proteins combined with subcellular fractionation.

**Fig. 1:**
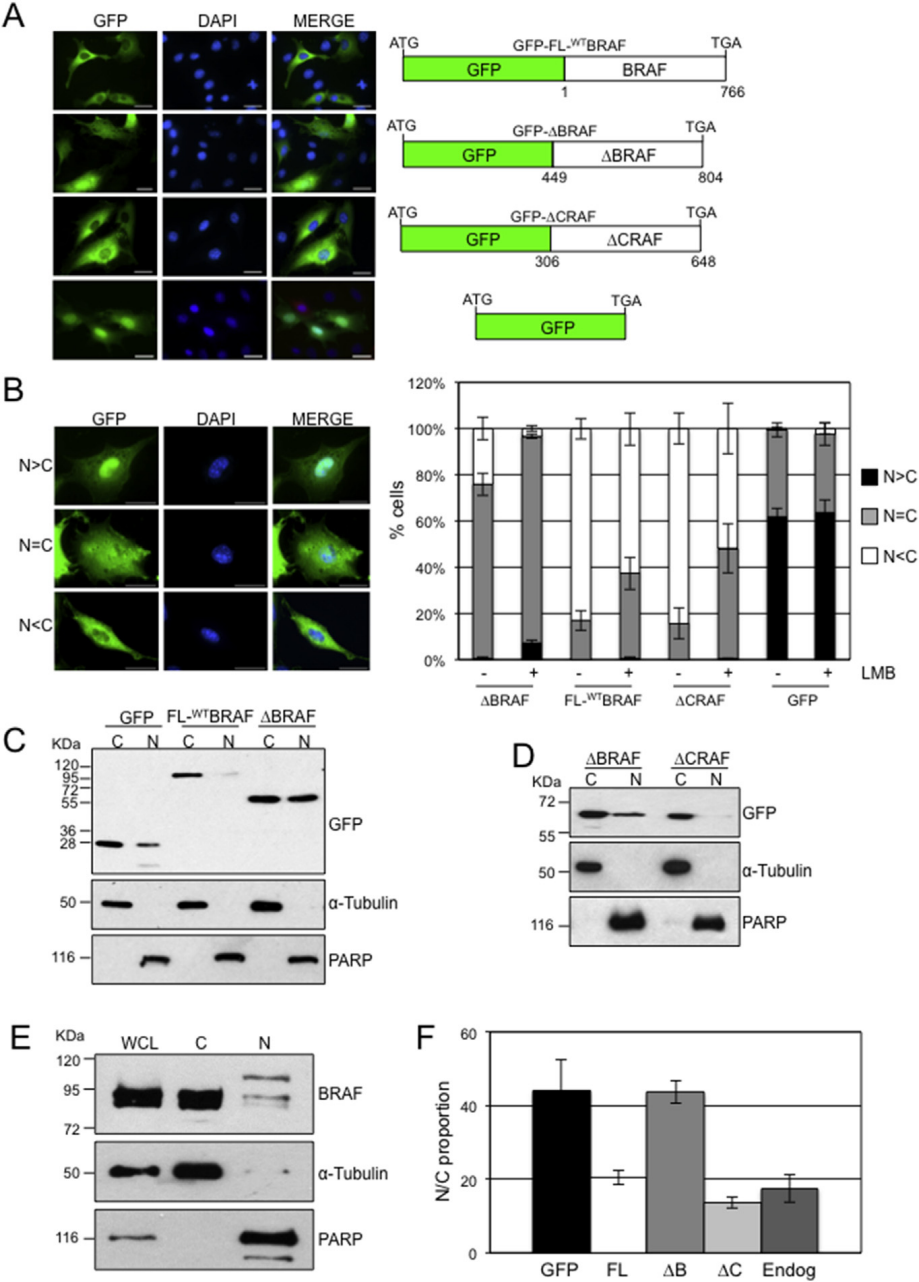
Localisation of GFP-RAF fusion proteins (A) GFP fluorescence imaging. NIH3T3 cells were transfected with vectors expressing GFP alone or the various RAF-GFP fusion proteins shown on the right. Cells were subjected to fluorescence imaging, generating images for GFP and DAPI, which were then merged. Scale bars, 50 μm. (B) Quantitation of GFP compartmentalisation. NIH3T3 cells were transfected with the vectors indicated and treated ± LMB. Following fluorescence microscopy, GFP fluorescence was categorized by the user as mostly nuclear (N > C), equally distributed (N=C) or mostly cytoplasmic (N < C). Over 200 cells were visualized for each transfection. Bar chart indicates mean (n = 3) ± SEM. Representative images of cells in each category are shown on the left. Scale bars, 50 μm. (C) Nuclear/cytoplasmic fractionation of GFP, GFP-FL-^WT^BRAF and GFP-ΔBRAF. NIH3T3 cells were transfected with the vectors indicated and subjected to subcellular fractionation 48 hours later. Fractions were analysed by western blot for GFP or with α-Tubulin or PARP to confirm purity of cytoplasmic and nuclear fractions. (D) Nuclear/cytoplasmic fractionation of GFP-ΔCRAF. NIH3T3 cells were transfected with the vectors indicated, fractions prepared and analysed by Western blot with antibodies for GFP, α-Tubulin or PARP. (E) Nuclear/cytoplasmic fractionation of endogenous BRAF. Whole cell Triton X-100 soluble lysates (WCL) of NIH3T3 cells were prepared as well as nuclear/cytoplasmic fractions and analysed with antibodies for BRAF, α-Tubulin or PARP. (F) Quantitation of western blot data. The Nuclear:Cytoplasmic (N:C) proportions were determined using Image J analysis of western blot signals. Density signals were adjusted for loading using signals for α-Tubulin (cytoplasmic loading) or PARP (nuclear loading). Values for the nuclear fractions were then divided by the total values for each sample. Data represent mean ± SEM (GFP, n = 8; GFP-FL-BRAF, n = 9; GFP-ΔBRAF, n = 10; GFP-ΔCRAF, n = 4; endogenous, n = 5).

cDNAs encoding either full-length BRAF (FL-^WT^BRAF) or the kinase domain of BRAF (ΔBRAF) were fused in-frame to a cDNA expressing GFP, generating N-terminal GFP tagged fusion cDNAs (Fig. 1A). The constructs were expressed in NIH3T3 cells and the staining patterns were evaluated. GFP-FL-^WT^BRAF showed mostly cytoplasmic staining but, unexpectedly, GFP-ΔBRAF demonstrated heterogeneous staining with evidence of both cytoplasmic and nuclear expression (Fig. 1A). A similar analysis with a fusion construct expressing GFP-ΔCRAF showed mostly cytoplasmic staining while a vector expressing the GFP tag alone showed mostly nuclear staining (Fig. 1A).

The staining pattern in each cell was classified into one of three categories: nuclear less than cytoplasmic (N < C), equally distributed (N=C) or nuclear greater than cytoplasmic (N > C) (Fig. 1B). Staining was quantified for each construct. For GFP-FL-^WT^BRAF and GFP-ΔCRAF, ∼80% of cells respectively demonstrated N < C staining, with ∼20% of cells having N=C staining and 0% of cells having N > C staining (Fig. 1B). With GFP-ΔBRAF, the proportion of cells with N=C staining increased to ∼75% and cells with N > C staining increased to ∼1%. Cells with N < C staining was reduced to ∼24% (Fig. 1B). Monomeric GFP showed ∼60% N > C staining.

For all GFP-RAF experiments, treatment of transfected cells with Leptomycin B (LMB), an inhibitor of Crm1-dependent nuclear export, significantly enriched the proportion of cells with nuclear staining. In particular, treatment of GFP-ΔBRAF-expressing NIH3T3 cells with LMB increased N=C cells to 90% and N > C cells to 7% while decreasing N < C cells to 3%. However, monomeric GFP did not show nuclear enrichment with LMB. This suggests a dynamic movement of the fusion proteins between the nucleus and cytoplasm (Fig. 1B), but not for monomeric GFP. GFP-ΔBRAF staining in the nucleus was shown to be real and intense by performing a Z series through a nuclear-stained cell using confocal microscopy imaging (Fig. S2).

To confirm the microscopy data, subcellular fractionation was performed. As with the fluorescence microscopy, monomeric GFP and GFP-ΔBRAF were robustly detected in cytoplasmic and nuclear fractions in NIH3T3 cells, whereas both GFP-FL-^WT^BRAF (Fig. 1C) and GFP-ΔCRAF (Fig. 1D) were detected at lower levels in this fraction. Consistent with the data for GFP-FL-^WT^BRAF, endogenous BRAF was also predominantly cytoplasmic although low levels of slower migrating isoforms were detected in the nucleus (Fig. 1E).

To gain a handle on the proportion of GFP-ΔBRAF in the nucleus, we assumed 100% nuclear localization for N > C cells, 100% cytoplasmic staining for N < C cells and 50:50 nuclear/cytoplasmic staining for N=C cells. Using these values, for GFP-ΔBRAF, ∼39% locates to the nucleus and this increases to ∼52% in the presence of LMB. For both GFP-FL-^WT^BRAF and GFP-ΔCRAF, ∼8% is located in the nucleus and this rises to ∼25% in the presence of LMB. Monomeric GFP showed ∼80% nuclear compartmentalisation. Determination of the Nuclear:Cytoplasmic (N:C) proportion by quantitation of western blots showed a broadly similar trend with mean values of 44% for GFP-ΔBRAF, 20% for GFP-FL-^WT^BRAF and 14% for GFP-ΔCRAF (Fig. 1F), although monomeric GFP showed less nuclear compartmentalisation by this method with a mean value of 44%. Quantitation of western blots, also generated a mean value of 18% for compartmentalisation of endogenous BRAF in the nucleus of NIH3T3 cells (Fig. 1F).

GFP fusion is used extensively as a method to study protein compartmentalisation and subcellular localisation. The main problem for nuclear localisation studies with GFP is that GFP translocates to the nucleus on its own by diffusion through the nuclear pore and, indeed, we detected the accumulation of monomeric GFP in the nucleus (Fig. 1A, B, C, F) consistent with previous observations [45]. Despite this, the fact that GFP-ΔBRAF and GFP-ΔCRAF both contain GFP and are of a similar size but have different distributions between the nucleus and cytoplasm is supportive of the RAF components being the major determinant of their compartmentalisation.

### 3.2 Localisation of hbER fusion proteins

**Fig. 2:**
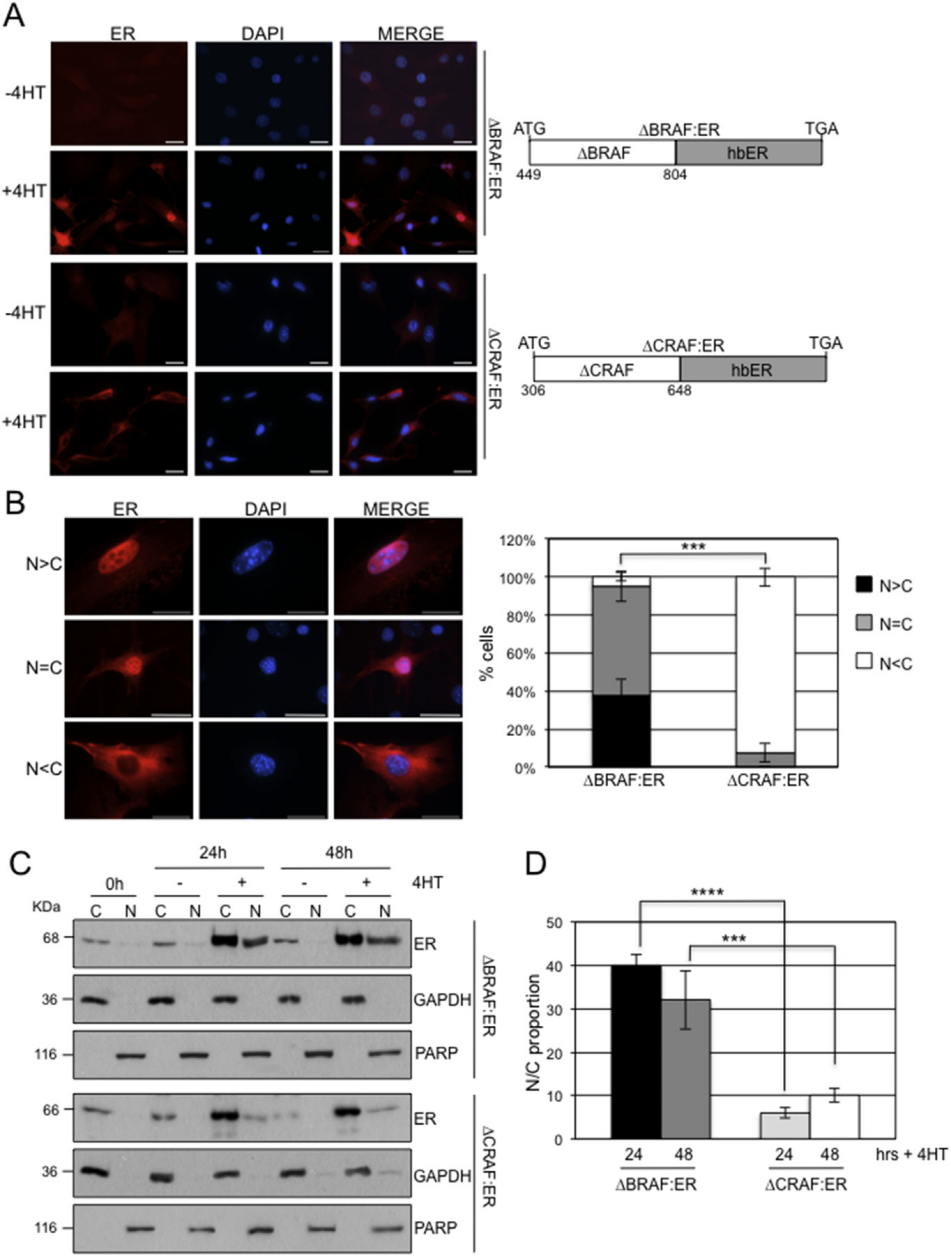
Localisation of ΔRAF:ER fusion proteins. (A) Schematics of the ΔBRAF:ER and ΔCRAF:ER vectors are shown on the right. NIH3T3 cells stably expressing these vectors were treated with or without 4-HT for 24 hours after which the cells were processed for immunofluorescence with an αER antibody. Representative images are shown on the left. Cells were counterstained with DAPI and the ER/DAPI images merged. Scale bars, 50 μm. (B) Quantitation of the subcellular compartmentalisation of ΔBRAF:ER and ΔCRAF:ER. RAF:ER-expressing cells treated with 4-HT for 24 hours were categorized as to whether the ER localisation was N > C, N=C or N < C. The bar chart indicates the mean of three experiments ± SEM. Representative images of cells within each category are shown on the left. Scale bars, 50 μm. (C) Fractionation of NIH3T3 cells expressing ΔBRAF:ER or ΔCRAF:ER. Nuclear and cytoplasmic fractions were prepared from NIH3T3 cells expressing ΔBRAF:ER or ΔCRAF:ER treated ± 4HT for the times indicated and analysed by western blot with the ER antibody. The purity of the fractions is shown by analysis with GAPDH (cytoplasmic marker) and PARP (nuclear marker). (D) Quantitation of western blot data. The N:C proportion of ER fusion proteins was determined using Image J analysis of Western blot signals. Data represent mean ± SEM (n = 3 for all samples).

To further rule out a role of the GFP tag, we assessed a different tag by using ΔBRAF:ER and ΔCRAF:ER, which represent fusions of the BRAF and CRAF kinase domains with the hormone binding domain of the oestrogen receptor (hbER) [38] (Fig. 2A). hbER-fusion proteins have been used extensively to investigate the downstream biological functions of a range of signaling proteins including MYC [46], ABL [47], and RAF [38, 48, 49]. Although the intact oestrogen receptor has the ability to localise to the nucleus, this function has been attributed to a Nuclear Localisation Signal (NLS) within the DNA binding domain and not the hormone binding domain [37, 50]. Control of chimeric hbER-fusion proteins is conferred by hormone-induced displacement of HSP90 binding leading to fusion protein stabilisation [51].

We used immunofluorescence with an ER antibody to determine the subcellular localisation of ΔBRAF:ER and ΔCRAF:ER stably expressed in NIH3T3 cells [38] before and after treatment with the anti-oestrogen 4-hydroxytamoxifen (4-HT). In the absence of 4-HT, there was weak staining reflecting the low levels of expression of the fusion proteins (Fig. 2A). In the presence of 4-HT, higher levels of stabilised proteins were observed with ΔCRAF:ER showing mostly cytoplasmic distribution and ΔBRAF:ER showing nuclear and cytoplasmic localisation (Fig. 2A). To obtain quantitative data, the staining pattern of each cell was categorised as above for Fig. 1B. We found a significant difference in the compartmentalisation of ΔBRAF:ER compared to ΔCRAF:ER following 4-HT treatment (Fig. 2B), with ΔBRAF:ER having significantly more N > C cells (∼38% compared to 0%) and N=C cells (∼57% compared to ∼8%) than ΔCRAF:ER with N < C cells reducing as a consequence (∼5% compared to ∼92%).

Fractionation was also undertaken and confirmed the observation that ΔBRAF:ER has a greater propensity to accumulate in the nucleus than ΔCRAF:ER following induction with 4-HT (Fig. 2C). Quantitation of western blot data showed 32–40% of ΔBRAF:ER in the nucleus as opposed to 6–10% for ΔCRAF:ER (Fig. 2D). These data are consistent with the results obtained for the GFP-tagged, exogenously-expressed proteins (Fig. 1).

### 3.3 Localisation of BRAF mutants detected in human cancers

**Fig. 3:**
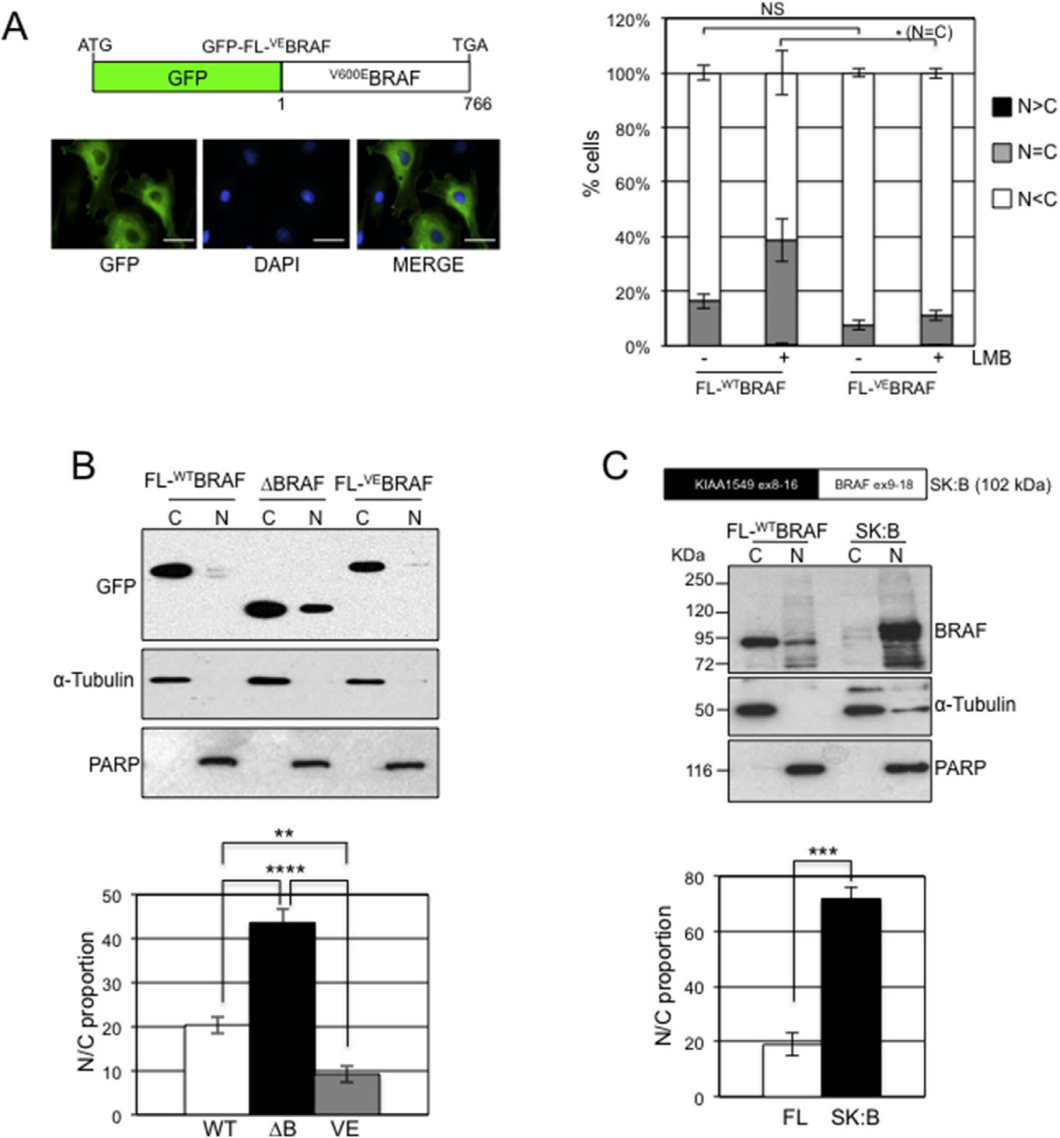
Localisation of oncogenic BRAF. (A) GFP fluorescence imaging of ^V600E^BRAF. NIH3T3 cells were transfected with vectors expressing either GFP-FL-^WT^BRAF as in Fig. 1B or GFP-^V600E^BRAF (schematic on the left). Cells were subjected to fluorescence imaging, generating images for GFP and DAPI, which were then merged. Representative images of GFP-^V600E^BRAF-expressing cells are shown on the left. Scale bars, 50 μm. GFP fluorescence was categorized as N > C, N = C or N < C. Over 200 cells were visualized for each transfection. The bar chart indicates mean (n = 3) ± SEM. The data for GFP-FL-^WT^BRAF are the same as that shown in Fig. 1B. (B) Fractionation of GFP-^V600E^BRAF. NIH3T3 cells were transfected with the vectors indicated and nuclear/cytoplasmic fractions were analysed with the antibodies indicated. Western blot data were quantified using Image J analysis to generate the N:C proportion as indicated in the bar chart. Data represent mean ± SEM (GFP-FL-^WT^BRAF, n = 9; GFP-ΔBRAF, n = 10; GFP-^V600E^BRAF, n = 4). The quantitative data for GFP-FL-^WT^BRAF and GFP-ΔBRAF are the same as that shown in Fig. 1F. (C) Fractionation of BRAF oncogenic fusion protein. NIH3T3 cells transiently expressing KIAA1549-BRAF (SK:B, shown above) or full-length, wild-type BRAF in pcDNA3.1 were subjected to fractionation and fractions were analysed with an antibody detecting the C-terminus of BRAF or with the α-Tubulin/PARP antibodies. Western blot data were quantified using Image J analysis to generate the N:C proportions presented in the bar chart. Data represent mean ± SEM (n = 3 for both FL-^WT^BRAF and SK:B).

We next investigated if the difference in distribution of full length versus N-terminally truncated BRAF also applied to mutant versions of BRAF that are detected in human cancers. A fusion construct was generated in which a cDNA for GFP was fused in-frame to a cDNA encoding human ^V600E^BRAF and this was expressed in NIH3T3 cells (Fig. 3A). Fluorescence microscopy was performed and the staining patterns quantitated (Fig. 3A). This showed 0% of cells with N > C staining, ∼8% with N=C staining and ∼92% with N < C staining. This distribution was not affected by LMB (Fig. 3A). Fractionation confirmed very low levels of GFP-^V600E^BRAF nuclear compartmentalisation (Fig. 3B), which was quantitated at ∼9% (Fig. 3B).

Although rare, over 50 different translocations involving the *BRAF* gene have been detected in human cancers, many associated with removal of the BRAF N-terminal regulatory domain and expression of novel fusion proteins with the BRAF C-terminal kinase domain [8]. We analysed the location of one such mutation involving exons 8–16 of the KIAA1549 gene fused to the C-terminal domain (exons 9–18) of BRAF (SK:B) reported in human pilocytic astrocytomas [33]. A vector expressing this fusion protein was transiently transfected into NIH3T3 cells and was found to show a greater level of accumulation in the nucleus than FL-^WT^BRAF as determined by fractionation and western blot analysis using a C-terminal antibody for BRAF (Fig. 3C). Indeed, quantitation of western blot data showed ∼70% of SK:B in the nuclear compartment, suggesting a contribution of the KIAA1549 translocation partner in its nuclear compartmentalisation.

Apart from the nuclear-localising potential of truncated BRAF, the subcellular localisation of each BRAF fusion protein could also be affected by the nature of the fusion partner, as found with SK:B (Fig. 3C). We performed cellular component ontology analysis of 55 gene partners for BRAF detected in human cancers and found 16 (29%) with the potential to locate to the nuclear compartment (Table S1). Thus, unlike ^V600E^BRAF, at least in some circumstances, fusion proteins in which the N-terminus of BRAF is replaced by a novel fusion partner have the potential to locate to the nucleus, whether this be mediated by the BRAF component or the fusion partner.

### 3.4 Canonical nuclear localisation signals are not present in ΔBRAF

**Fig. 4:**
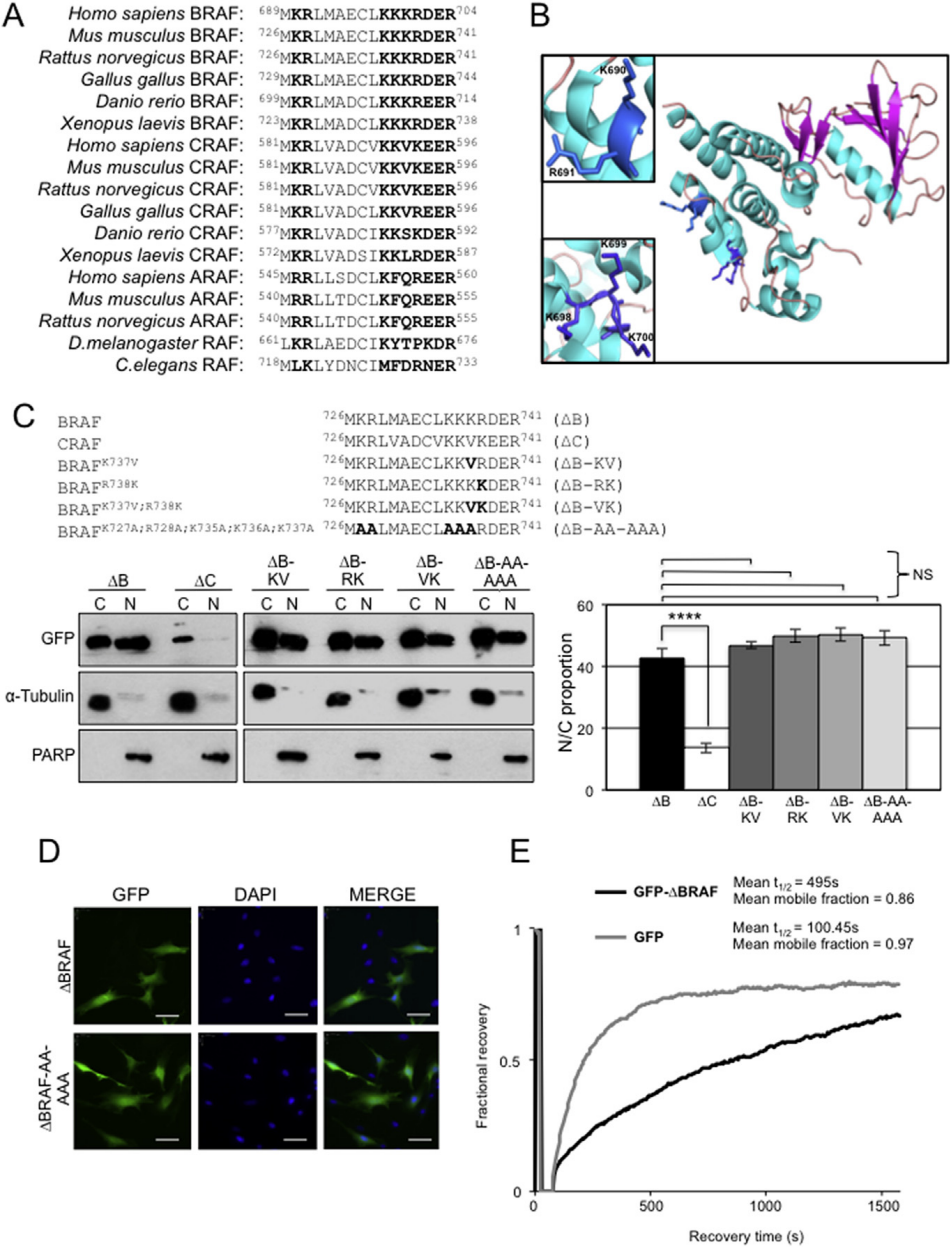
GFP-ΔBRAF nuclear import is independent of a classical NLS. (A) Comparison of the amino acid sequences of a putative bipartite NLS within BRAF and RAF homologues from the indicated species. Sequences were taken from Ensembl (www.ensembl.org/index.html). (B) Ribbon diagram of the putative bipartite NLS within human BRAF. Expanded views of the exposed motifs at basic residues 690-691 (top expanded view) and 698-704 (bottom expanded view) are shown. (C) Mutation of the putative bipartite NLS in GFP-ΔBRAF. Four different mutants were generated within the putative NLS sequence at residues 726-741 of GFP-ΔBRAF. These vectors, along with GFP-ΔBRAF and GFP-ΔCRAF were transfected into NIH3T3 cells and nuclear and cytoplasmic fractions prepared. Western blots were analysed with the antibodies indicated. Western blot data were quantified using Image J analysis to generate the N:C proportion for the fusion proteins indicated in the bar chart on the right. Data represent mean ± SEM (GFP-ΔBRAF, n = 10; GFP-ΔCRAF, n = 4; all mutants, n = 3). The quantitative data for GFP-ΔBRAF and GFP-ΔCRAF are the same as that shown in Fig. 1F. (D) Representative fluorescence microscopy images of GFP-ΔBRAF and GFP-ΔBRAF carrying the AA-AAA mutations. Scale bars, 50 μm. (E) FRAP analysis of GFP-ΔBRAF. NIH3T3 cells transfected with vectors expressing either GFP-ΔBRAF or monomeric GFP were subjected to FRAP. Mean mobile fractions and recovery half times are shown. Data for individual cells are shown in Fig. S3.

The fact that LMB enriches ΔBRAF in the nucleus (Fig. 1B) indicates that the protein can shuttle in and out of this compartment. Unlike MEK and ERK [24], mammalian ΔBRAF does not possess a purported NTS with the S/T-P-S/T sequence and, in contrast to MEK, ΔBRAF also does not possess a Crm1-dependent leucine-rich canonical NES. To search for a canonical NLS we used the PSORT algorithm (https://psort.hgc.jp), which searches for sequences homologous to the classical NLS of the SV40 large T antigen. This analysis identified a potential bipartite NLS at residues 689-704 of human BRAF that is conserved in several including *D. rerio* and *X. laevis* (Fig. 4A). The contiguous nature of basic amino acids in this sequence is disrupted in CRAF and ARAF as well as in the RAF homologues of *C. elegans* and *D. melanogaster* (Fig. 4A). The 3D structure of the kinase domain shows the putative bipartite NLS of BRAF lies in a loop region, exposed to potential solvent and binding partners (Fig. 4B).

To examine whether this putative NLS sequence is functional, we created two different types of mutations in mouse GFP-ΔBRAF: a) K737V and/or R738K mutation in order to create sequences analogous to those in CRAF; b) combined K727A, R728A, K735A, K736A, K737A mutations to disrupt contiguous basic amino acids. None of these mutation strategies altered GFP-ΔBRAF compartmentalisation as determined by fractionation following expression in NIH3T3 cells (Fig. 4C, D), suggesting that the mechanisms governing import of ΔBRAF do not conform to the classical NLS model.

To investigate mobility of ΔBRAF in real time, FRAP was performed for GFP-ΔBRAF or monomeric GFP in NIH3T3 cells after photobleaching of the nuclei of GFP-expressing cells. There was rapid, almost complete nuclear recovery of monomeric GFP indicating that GFP moves freely between compartments (Fig. 4E and Fig. S3). This is consistent with the observation that GFP localisation is not sensitive to LMB treatment (Fig. 1B). GFP-ΔBRAF showed slower recovery and with a lower mobile fraction (Fig. 4E and Fig. S3). These data show that ΔBRAF-GFP movement to the nucleus was more complex than monomeric GFP and possibly involves the regulation of multiple populations with differing translocation rates.

### 3.5 BRAF nuclear compartmentalisation is suppressed by the CR1 domain

**Fig. 5:**
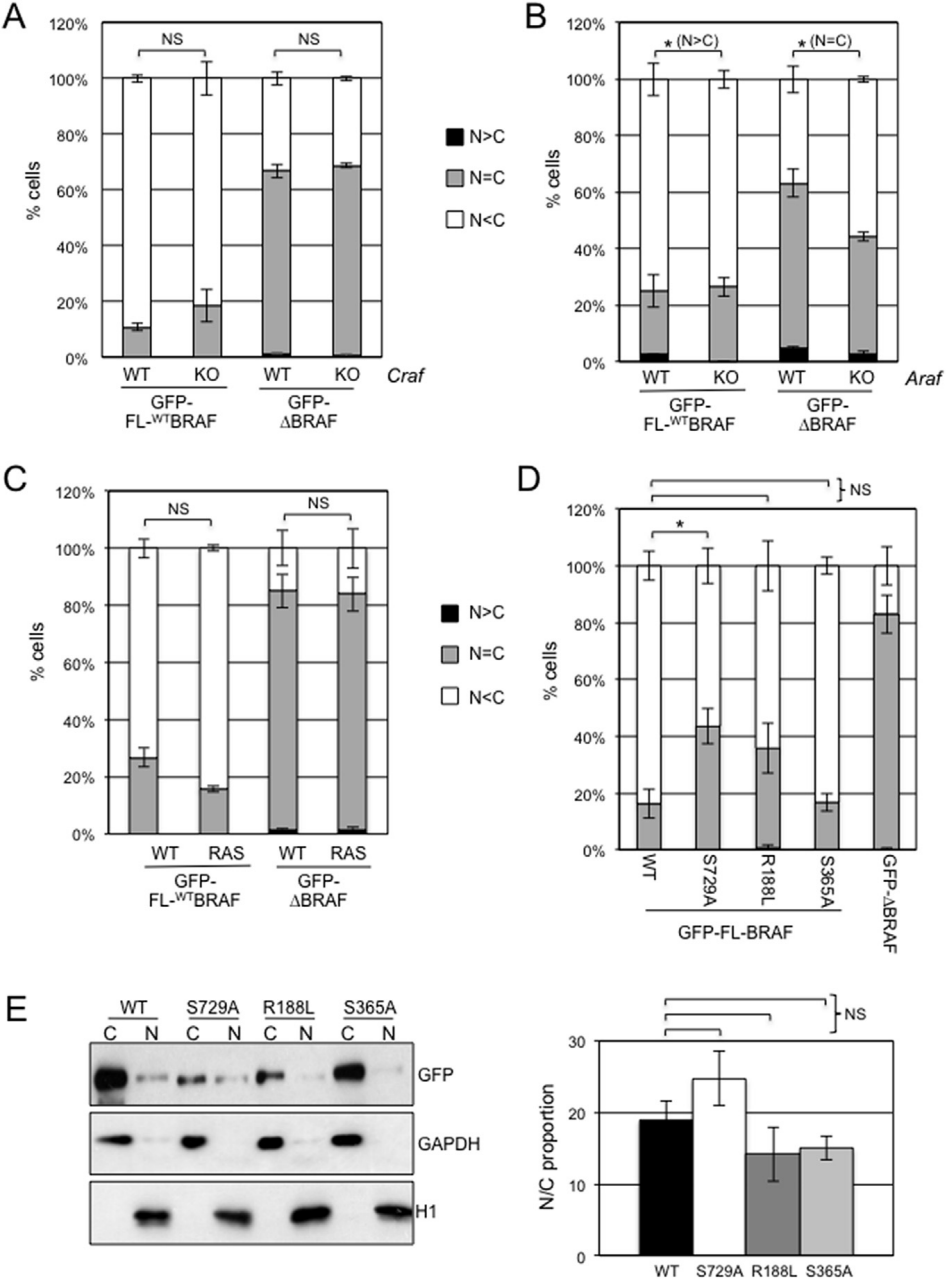
Effect of CRAF, ARAF, ^G12D^KRAS and 14-3-3 on GFP-BRAF localisation. (A) GFP-FL-^WT^BRAF or GFP-ΔBRAF expression in *Craf* KO or wild-type MEFs followed by fluorescence microscopy quantitation. (B) GFP-FL-^WT^BRAF or GFP-ΔBRAF in *Araf* KO or wild-type MEFs followed by fluorescence microscopy quantitation. (C) GFP-FL-^WT^BRAF or GFP-ΔBRAF expression in wild-type or KRAS^G12D^ MEFs followed by fluorescence microscopy quantitation. (D) GFP-FL-BRAF with or without the R188L, S729A or S356A mutations or GFP-ΔBRAF expressed in NIH3T3 cells followed by fluorescence microscopy quantitation. For (A–D), cells were categorized as in Fig. 1B and bar charts show mean (n = 3) ± SEM. In (A–C), three different MEFs of each genotype were examined. (E) Cells from (D) were subjected to nuclear/cytoplasmic fractionation and western blots analysed with antibodies against GFP or with GAPDH or Histone H1 to confirm purity of fractions. Western blot data were quantified using Image J analysis to generate the N:C proportion indicated in the bar chart on the right. Data represent mean ± SEM (n = 3 for each vector).

The fact that GFP-ΔBRAF has the potential to accumulate in the nucleus whereas GFP-FL-^WT^BRAF does not accumulate to the same extent suggests possible tethering of full length BRAF in the cytoplasm through its N-terminal domain. RAF proteins are known to hetero-dimerise [52] and therefore we tested the roles of CRAF and ARAF in BRAF compartmentalisation. There was no difference in the compartmentalisation of GFP-FL-^WT^BRAF or GFP-ΔBRAF following expression in *Craf* knockout MEFs (Fig. 5A) while, in *Araf* knockout MEFs, there was a small but significant decrease in nuclear compartmentalisation of both fusion proteins (Fig. 5B). However, overall, these data identify no role for heterodimerisation in the tethering of the full-length protein in the cytoplasm.

We also examined a role of RAS by expressing GFP-FL-^WT^BRAF and GFP-ΔBRAF in MEFs expressing oncogenic ^G12D^KRAS and found that there was no significant difference in compartmentalisation compared to wild-type MEFs (Fig. 5C). Consistently, there was no significant difference in the compartmentalisation of GFP-FL-^R188L^BRAF, which bears a mutation preventing RAS binding as assessed by both fluorescence microscopy (Fig. 5D) and fractionation (Fig. 5E). Creation of mutations in the 14-3-3 binding residue at S729A in GFP-FL-BRAF showed a small but significant increase in nuclear compartmentalisation as assessed by fluorescence microscopy (Fig. 5D) but this was not validated by fractionation (Fig. 5E). There was also no significant difference with respect to mutation of the S365 14-3-3 binding site (Fig. 5D, E).

It should be noted that, throughout our experiments, we observed some variability in the level of nuclear/cytoplasmic compartmentalisation of both GFP-FL-^WT^BRAF and GFP-ΔBRAF in NIH3T3 cells (Fig. 1B) compared to the wild-type MEFs used in Fig. 5. We compared the pattern of staining more directly but found there was no significant difference between the cell types except for slightly increased nuclear staining of GFP-ΔBRAF in Araf^WT^ cells compared to NIH3T3 cells (Fig. S4). The reason for this is not clear but may be reflective of the cellular origin of these different fibroblastic cell lines.

**Fig. 6:**
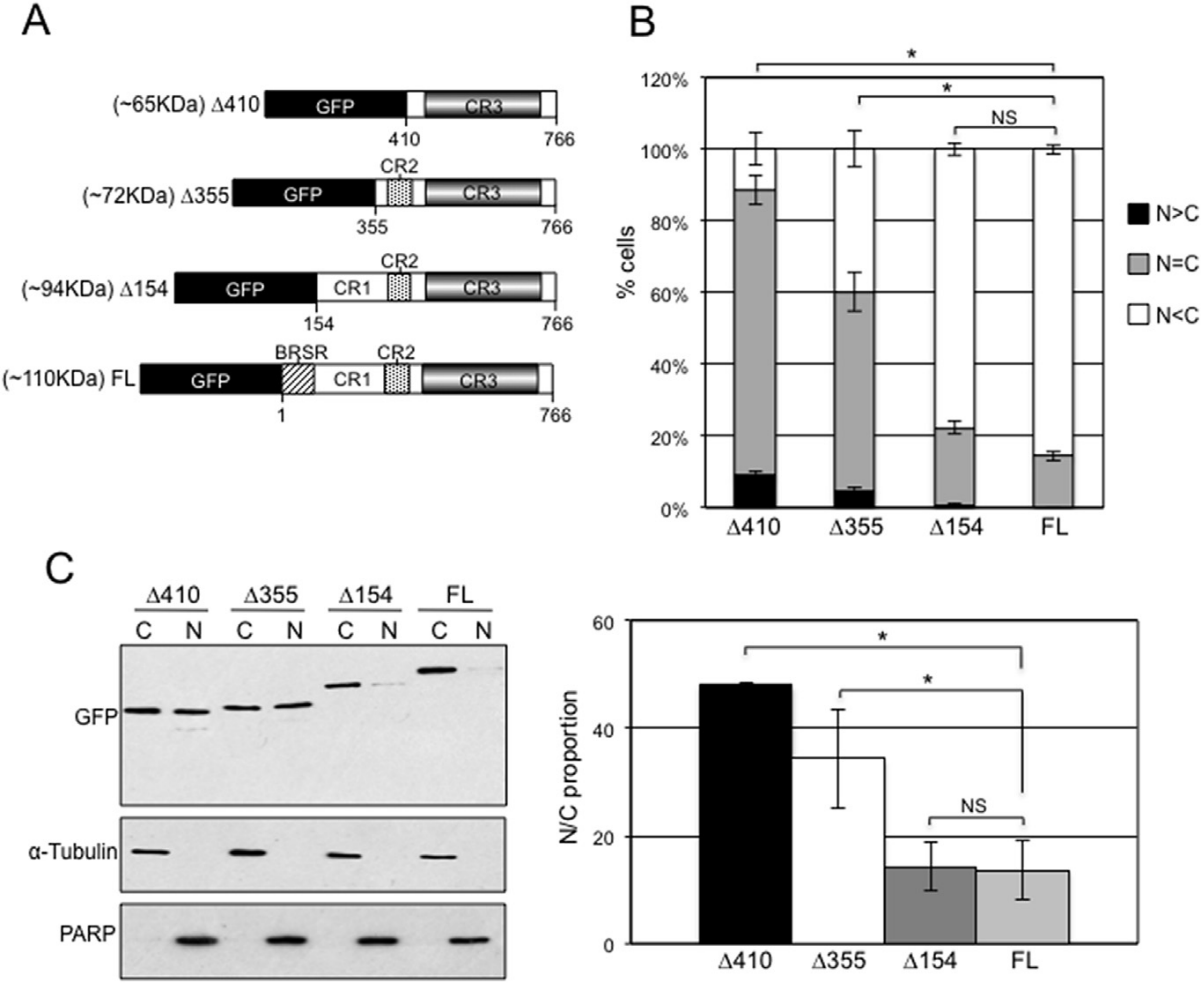
GFP-ΔBRAF nuclear compartmentalisation is suppressed by the CR1 domain. (A) Diagrams indicate the full length and truncated versions of human BRAF-GFP generated. (B) The vectors from (A) were transfected into NIH3T3 cells and cells were categorised as in Fig. 1B. Bar chart indicates mean (n = 3) ± SEM. (C) Cells from (B) were also subjected to nuclear/cytoplasmic fractionation, which were analysed by western blot with the antibodies indicated. Western blot data were quantified using Image J analysis to generate the N:C proportion indicated in the bar chart on the right. Data represent mean ± SEM (n = 3 for each vector).

To investigate the role of the N-terminus in tethering full-length BRAF in the cytoplasm, we created serial truncations of human full-length ^WT^BRAF (Fig. 6A) and found that increasing the size of the deletion from the N-terminus (∼110 KDa to ∼65 KDa including GFP) progressively increased BRAF nuclear accumulation with both the CR1 domain and BRAF-specific region (BRSR) suppressing BRAF nuclear compartmentalisation (Fig. 6B, C). These experiments also confirm that human GFP-ΔBRAF (Δ410) has a similar subcellular distribution to the equivalent mouse GFP-ΔBRAF (Fig. 1A, B, C, D).

In summary, although hetero-dimerisation and RAS binding do not regulate nuclear compartmentalisation of the full-length BRAF protein, the BRSR and CR1 are important in its cytoplasmic retention.

### 3.6 Analysis of phosphorylated MEK and ERK

**Fig. 7:**
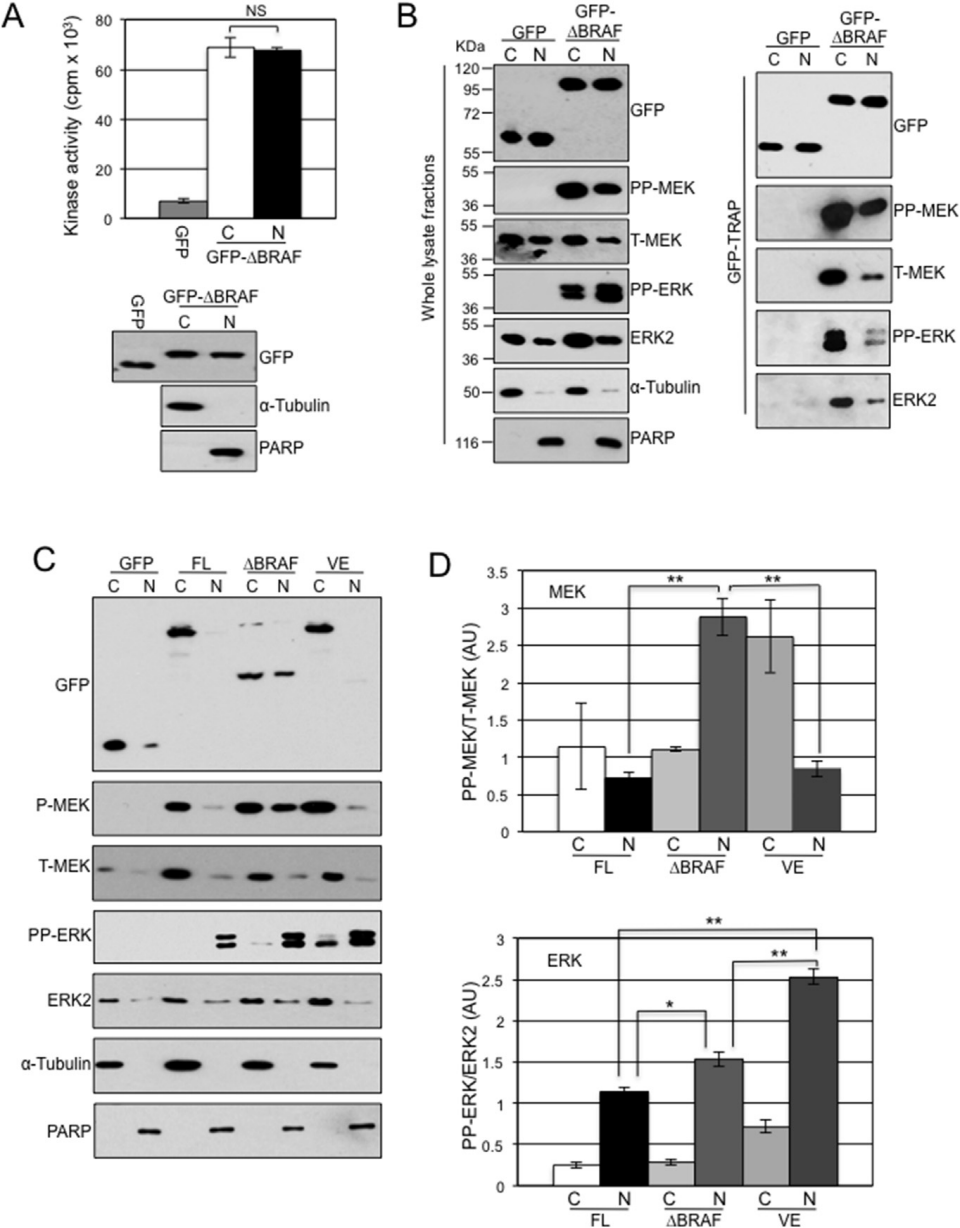
Localisation of phosphorylated MEK and ERK. (A) Nuclear GFP-ΔBRAF has kinase activity towards the MEK-ERK pathway. NIH3T3 cells were transfected with vectors expressing GFP or GFP-ΔBRAF. Whole cell lysates were prepared from the GFP-transfected cells while nuclear/cytoplasmic fractions were prepared from the GFP-ΔBRAF transfected cells. GFP expression levels and purity of fractions are indicated by the western blots on the bottom. Samples were subjected to immunoprecipitation for GFP and kinase cascade assays were performed. Data shows mean ± SD of three independent experiments. (B) Nuclear GFP-ΔBRAF binds to phosphorylated and non-phosphorylated MEK and ERK. NIH3T3 cells were transfected with vectors expressing GFP or GFP-ΔBRAF, nuclear/cytoplasmic fractions were prepared and analysed for GFP, α-Tubulin or PARP as well as components of the MAPK pathway. A portion of the fractions was subjected to GFP-TRAP immunoprecipitation and immunoprecipitates were subjected to western blot analysis with the antibodies indicated. (C) Phosphorylated MEK and ERK accumulate in the nucleus of cells expressing GFP-ΔBRAF. NIH3T3 cells were transfected with the vectors indicated. Nuclear and cytoplasmic fractions were prepared and analysed by western blot with the indicated antibodies. (D) Quantitation of western blot data from (C). Image J analysis was used to determine density signals of each band on western blots which were adjusted for loading using signals for α-Tubulin (cytoplasmic loading) or PARP (nuclear loading). In the upper bar chart, PP-MEK value were adjusted for total MEK values while in the lower bar chart PP-ERK values were adjusted for ERK2 values. The data are presented as Arbitrary Units (AU) and represent mean ± SEM (n = 3 for each condition).

While the above data indicate a role for the N-terminus in preventing BRAF nuclear accumulation, this may also be attributable to the suppressive effect of this domain on BRAF kinase activity [53] and therefore we investigated the link between downstream MEK-ERK activation and GFP-BRAF compartmentalisation. Using fractionation followed by immunoprecipitation and kinase cascade assays, we found that nuclear GFP-ΔBRAF had similar levels of kinase activity towards MEK-ERK as cytoplasmic GFP-ΔBRAF (Fig. 7A) and could bind to phosphorylated and non-phosphorylated forms of MEK and ERK (Fig. 7B). As a control, analysis of GFP alone showed negligible kinase activity and no binding to phosphorylated or non-phosphorylated MEK/ERK (Fig. 7A, B).

We monitored the compartmentalisation of MEK and ERK following expression of GFP, GFP-ΔBRAF, GFP-FL-^WT^BRAF and GFP-FL-^VE^BRAF in NIH3T3 cells using fractionation. In all cases, non-phosphorylated MEK1/2 and ERK2 were predominantly cytoplasmic and this distribution was not noticeably different between samples (Fig. 7C). As expected from the data shown in Fig. 7B, monomeric GFP did not induce detectable MEK or ERK phosphorylation (Fig. 7C). In contrast, all forms of GFP-BRAF induced accumulation of phosphorylated ERK (PP-ERK) in the nucleus (Fig. 7C), although the levels of nuclear PP-ERK were significantly higher in the cells expressing the oncogenic mutants ΔBRAF and ^V600E^BRAF (Fig. 7C, D). Intriguingly, although all GFP-BRAF forms induced MEK phosphorylation (PP-MEK), its distribution was noticeably different in that it accumulated in the nucleus to a significantly greater extent in the case of GFP-ΔBRAF than either GFP-FL-^WT^BRAF or GFP-FL-^VE^BRAF (Fig. 7C, D). This is a surprising result given that phosphorylated and non-phosphorylated forms of MEK are normally actively exported out of the nucleus through its NES [29].

**Fig. 8:**
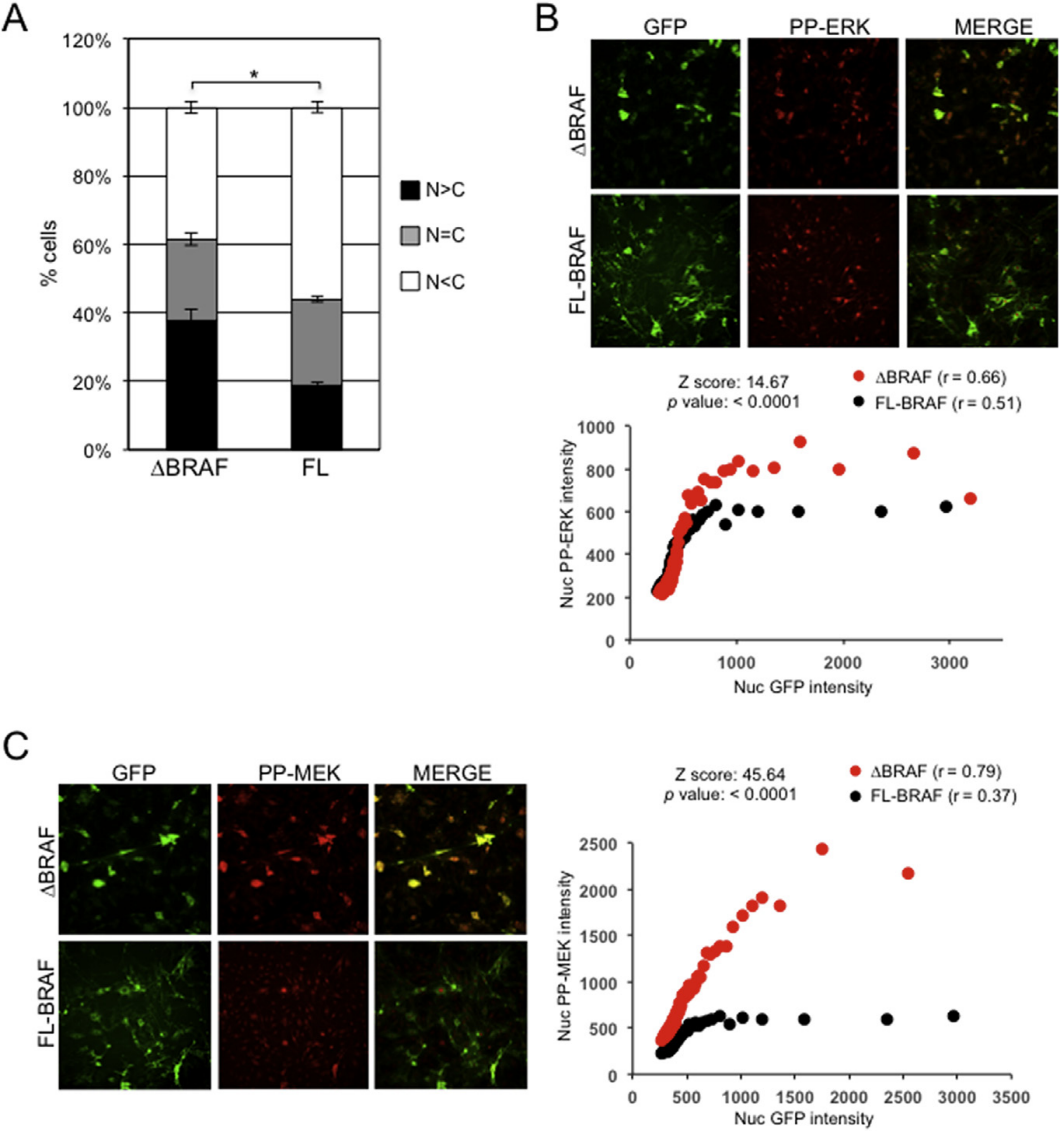
High Content Microscopy (HCM). (A) Localisation of BRAF-GFP using HCM. NIH3T3 cells expressing either GFP-ΔBRAF or GFP-FL-^WT^BRAF were treated with LMB for 3 hours and then subjected to HCM analysis. GFP staining was categorised into one of three categories as indicated where N > C represents >1.5 class nuclear staining, N=C represents 1–1.5 class nuclear staining and N < C represents <1 class nuclear staining. Mean values ± SEM are shown. (B) Localisation of PP-ERK. NIH3T3 cells expressing GFP-FL-^WT^BRAF or GFP-ΔBRAF were treated with LMB for 3 hours, immunostained for PP-ERK1/2 and subjected to HCM. Representative digital images are shown. Cells were sorted into bins as described in Materials andMethods. The data are population averages (in AFU) from 3 repeat experiments, each with duplicate adenovirus infections and 500–600 cells per infection. Correlation coefficients (r) were determined for each dataset, converted to Z scores and compared to generate the *p* value indicated. (C) Localisation of PP-MEK. NIH3T3 cells expressing GFP-FL-^WT^BRAF or GFP-ΔBRAF were treated with LMB for 3 hours, immunostained for PP-MEK and analysed by HCM. Representative digital images are shown. Cells were sorted into bins as described in Materials andMethods. The data in the graph are population averages (in AFU) from 3 repeat experiments, each with duplicate adenovirus infections and 500–600 cells per infection. Correlation coefficients (r) were determined for each dataset, converted to Z scores and compared to generate the *p* value indicated.

To further examine the link between GFP-BRAF compartmentalisation and phosphorylated MEK-ERK, high content microscopy (HCM) was utilised to allow the monitoring of thousands of cells. Initial analysis confirmed strong GFP-ΔBRAF nuclear compartmentalisation and lower levels of GFP-FL-^WT^BRAF in the nucleus following LMB treatment of the cells (Fig. 8A). By utilising the same method as that described above for determining the proportion of GFP-BRAF in the nucleus (i.e. 100% nuclear localization for N > C cells, 100% cytoplasmic staining for N < C cells and 50:50 nuclear/cytoplasmic staining for N=C cells), ∼50% of GFP-ΔBRAF was found to be nuclear localised and ∼30% of GFP-FL-^WT^BRAF. These values are consistent with the LMB-treated values determined from Fig. 1B.

We then undertook simultaneous PP-ERK or PP-MEK immunofluorescence (Fig. 8B, C). This revealed that nuclear PP-ERK and PP-MEK levels were typically higher for GFP-ΔBRAF than GFP-FL-^WT^BRAF and this distinction was more pronounced for PP-MEK, consistent with the fractionation data (Fig. 7C, D). Both nuclear GFP-ΔBRAF and nuclear GFP-FL-BRAF showed positive correlations with nuclear PP-ERK (Fig. 8B; correlation coefficients of 0.66 and 0.51 respectively) and with nuclear PP-MEK (Fig. 8C; correlation coefficients of 0.79 and 0.37 respectively). There were statistically significant differences between the correlation coefficients of GFP-ΔBRAF and GFP-FL-BRAF for both PP-ERK and PP-MEK, but this was particularly marked for PP-MEK (Z score of 45.64 compared to 14.67 for PP-ERK). In contrast, mock and GFP-transfected cells showed negligible PP-MEK or PP-ERK staining by immunofluorescence analysis (Fig. S5). Thus, over a broad range of expression levels, truncation of BRAF caused a marked increase in nuclear PP-MEK and a more modest increase in nuclear PP-ERK, consistent with the fractionation data (Fig. 7C, D). Similar relationships were seen in control cells and in cells stimulated for 15 or 120 min with PDGF (Fig. S6).

We attempted to use other methods to manipulate MEK-ERK activity, particularly BRAF and MEK inhibitors. However, interpretation of these experiments was complicated by the fact that we observed altered BRAF expression levels in all compartments (unpublished observations), presumably due to feedback regulation of BRAF protein stability by the ERK pathway as previously reported [54, 55].

## 4 Discussion

An overwhelming body of evidence has shown that the BRAF protein kinase, in its inactive conformation, is located in the cytoplasm as part of a multiprotein complex containing 14-3-3 adaptor/scaffold proteins and heat-shock protein chaperones [12]. Following upstream stimulation by ligand-activated receptor tyrosine kinases, BRAF forms a network of interacting kinases including ARAF, CRAF and KSR1/2 that bind to RAS.GTP at the plasma membrane, leading to RAF phosphorylation of MEK1/2, which in turn activates ERK1/2 [52]. Despite this widely-accepted model of BRAF regulation, a number of other subcellular locations have been reported for BRAF [27, 28, 29, 30, 31, 32, 56]. Here, using fluorescence imaging and fractionation of tagged, exogenous proteins expressed in NIH3T3 cells, we provide evidence in support of the location of wild-type and oncogenic forms of full length BRAF in the cytoplasm of the cell. However, we also show that N-terminally deleted forms of tagged BRAF can accumulate in the nucleus when over-expressed in NIH3T3 cells, in contrast to N-terminally deleted CRAF. This difference is observed regardless of the type of tag used to monitor the truncated proteins.

N-terminally truncated forms of BRAF and CRAF were originally identified as the transforming proteins expressed within mouse and avian oncogenic retroviruses [1]. Extensive biochemical studies on these truncated proteins showed they carry constitutive serine/threonine protein kinase activity with the ability to activate the downstream MEK/ERK pathway [1]. In particular, RAF-hbER fusion proteins expressed in NIH3T3 have been widely used as tools to unravel downstream RAF effects [38, 48, 49]. Our finding that ΔBRAF and ΔCRAF have different distributions between the nuclear and cytoplasmic compartments (Figs. 1 and 2) should be taken into consideration alongside these data as, clearly, this could impact on underpinning mechanisms. Indeed, the observation that ΔBRAF:ER has stronger activity towards the MEK/ERK pathway than ΔCRAF:ER [38] may be related to their different distributions within the cell.

The differences in the compartmentalisation of GFP-tagged, N-terminally truncated as opposed to full-length BRAF is also interesting and potentially has implications for cancers bearing single nucleotide variants as opposed to structural variants of BRAF. However, as a next step, it will be important to track the expression of endogenous RAF proteins in normal and cancer cells, and under different conditions, in order to confirm the findings from the over-expression studies. Development of a specific BRAF antibody for immunofluorescence will be important for this and/or the introduction of a protein tag into the endogenous RAF genes using genetic modification.

A widely accepted paradigm for nuclear import is that small proteins of <40KDa can freely translocate through the Nuclear Pore Complex (NPC) while proteins of a larger mass use an energy-dependent mechanism involving selective transport machinery. The ΔBRAF-GFP and ΔBRAF:ER fusion proteins used in this study have molecular masses of ∼65KDa and ∼68KDa respectively, suggesting they are too large to transit to the nucleus by diffusion, while monomeric GFP at ∼27KDa is able to transit by diffusion. Consistently, the accumulation of the larger KIAA1549-BRAF fusion protein (∼102KDa) in the nucleus argues against a diffusion mechanism (Fig. 3C). However, our data also exclude a role of energy-dependent selective transport that relies on the classical NLS-dependent import mechanism in the nuclear import of ΔBRAF (Fig. 4). Therefore, the mode of nuclear import of ΔBRAF is currently not clear. The fact that ΔBRAF accumulates in the nucleus in the same cells that have also accumulated PP-MEK and PP-ERK in the nucleus (Figs. 7 and 8) and that nuclear ΔBRAF can bind to PP-MEK/PP-ERK in co-immunoprecipitation studies (Fig. 7B) raises the possibility that they are imported as a complex.

ERK translocation occurs through both passive and facilitated processes, with some evidence showing that non-phosphorylated monomeric ERK is translocated by diffusion while phosphorylated dimeric ERK requires energy for nuclear import [17, 57]. The lack of a conventional NLS sequence in ERK and importinαβ binding has questioned the mechanisms associated with its active nuclear import, although there is some evidence to show that phosphorylation of an SPS motif in the ERK kinase insertion domain, allows for interaction with importin7 and nuclear import [24, 58]. Critical to passive and facilitated nuclear import of ERK is its interaction with NPC proteins, the nucleoporins [59]. Not only is this important for ERK nuclear entry, but ERK phosphorylation of FG-contain nucleoporins NUP50, NUP153 and NUP214 has been shown to regulate their affinity for importinβ, thus inhibiting nuclear protein import [59]. It will be interesting to further examine the involvement of ERK in ΔBRAF nuclear import and whether ΔBRAF is also involved in the regulation of generalised nuclear protein import by ERK.

Although MEK is also translocated to the nucleus through passive and facilitated mechanisms, it is rarely detected in the nucleus due to its possession of a NES, which allows for its rapid nuclear export. The fact that PP-MEK accumulates in the nucleus in the presence of ΔBRAF suggests interference with the nuclear export of PP-MEK, possibly by ΔBRAF acting as an anchor for MEK in the nucleus or by interfering with its NES. The localisation of PP-MEK in the nucleus is unusual and may have physiological significance. Nuclear accumulation of phosphorylated MEK has been previously linked with MEK acetylation [60], prolongation of MEK-ERK activation [61], enhancement of colon carcinogenesis [61] and the generation of polyploidy [62]. It will be important to examine if N-terminally truncated BRAF, as detected in several human cancers, is linked with phosphorylated MEK nuclear accumulation and whether this is associated with any of the key biochemical and cellular characteristics previously linked with aberrant MEK localisation.

Our data also exclude the involvement of RAF heterodimerisation and RAS interaction (Fig. 5A, B, C) in tethering full length BRAF in the cytoplasm but we show the importance of the BRSR and CR1 domain (Fig. 6). Although we did not detect a canonical NES in the BRAF protein sequence, we cannot as yet rule out a role of a non-canonical NES in the location of full-length BRAF. The RAF N-terminal domain auto-inhibits RAF activity through intramolecular interaction with the kinase domain [53], suggesting a possible role of deregulated BRAF catalytic activity in BRAF distribution within the cell. However, this cannot be the only contributing factor as the constitutively active ΔBRAF and ^V600E^BRAF mutants have different locations. Clearly, further studies are required to unravel the mechanisms by which ΔBRAF translocation to the nucleus becomes possible and the role of BRAF kinase activity in this. So far, it has not been possible to use BRAF or MEK inhibitors since these are known to influence BRAF protein stability [55, 63].

In summary, our data provide evidence for the accumulation of over-expressed, exogenous, N-terminally truncated forms of BRAF in the nucleus of the cell and that this is accompanied by the accumulation of phosphorylated MEK and ERK in the nucleus. Clearly, further studies will be needed to validate these data by tracking the intracellular distribution of endogenous proteins in normal and cancer cells. Nevertheless, our data is important to consider in the aetiology of cancers expressing N-terminally deleted forms of BRAF including splice variants that arise in tumours with acquired resistance to the BRAF inhibitor vemurafenib [9].

## Declarations

### Author contribution statement

Fiona Hey, Catherine Andreadi, Catherine Noble, Bipin Patel, Hong Jin, Tamihiro Kamata, Kees Straatman: Performed the experiments; Analyzed and interpreted the data.

Jinli Luo: Analyzed and interpreted the data.

Kathryn Balmanno: Performed the experiments.

David T. W. Jones, V. Peter Collins: Contributed reagents, materials, analysis tools or data.

Simon J. Cook, Christopher J. Caunt, Catrin Pritchard: Conceived and designed the experiments; Analyzed and interpreted the data; Wrote the paper.

### Funding statement

This study was supported by Worldwide Cancer Research (Grant number: 09-0412) and Cancer Research UK (Grant number: C1362/A6969).

### Competing interest statement

The authors declare no conflict of interest.

### Additional information

No additional information is available for this paper.
