# Supplementary material for: Over-expressed, N-terminally truncated BRAF is detected in the nucleus of cells with nuclear phosphorylated MEK and ERK
Source: Heliyon. 2018 Dec 20;4(12):e01065. doi: 10.1016/j.heliyon.2018.e01065 (PMC6304467; doi:10.1016/j.heliyon.2018.e01065)
Supplement: Supplementary Information [file mmc1.pdf]

Over-expressed, N-terminally truncated BRAF is detected in the nucleus of cells with nuclear phosphorylated MEK and ERK

Fiona Hey, Catherine Andreadi, Catherine Noble, Bipin Patel, Hong Jin, Tamihiro Kamata, Kees Straatman, Jinli Luo, Kathryn Balmanno, David T. W. Jones, V. Peter Collins, Simon J. Cook, Christopher J. Caunt, Catrin Pritchard

SUPPLEMENTARY INFORMATION

SUPPLEMENTARY METHODS
Cell culture
Immunofluorescence analysis

SUPPLEMENTARY TABLE

Table S1. Cellular component ontogeny analysis of BRAF translocation partners detected in human cancers.

SUPPLEMENTARY FIGURES

Figure S1. Immunofluorescence analysis of endogenous BRAF.

Figure S2. Confocal analysis of GFP-ΔBRAF.

Figure S3. Individual FRAP analysis of GFP and GFP-ΔBRAF.

Figure S4. Comparison of GFP-BRAF distributions in different cell types.

Figure S5. Immunostaining for phosphorylated MEK and ERK in control transfected cells.

Figure S6. Phosphorylated MEK and ERK localisation following PDGF stimulation using High Content Microscopy.

IMAGES OF ORIGINAL WESTERN BLOTS

Additional References:

Latyasheva NS and Babu MM (2016) Discovering and understanding oncogenic gene fusions through data intensive computational approaches. Nucl Acids Res 44: 4487-4503.

Ross JS, Wang K, Chmielecki J, Gay L, Johnson A, Chudnovsky J, Yelensky R, Lipson D, Ali SM, Elvin JA, Vergilio J-A, Roels S, Miller VA, Nakamura BN, Gray A, Wong MK, Stephens PJ (2016) The distribution of BRAF gene fusions in solid tumors and response to targeted therapy. Int J Cancer 138: 881-890.

Urakami K, Shimoda Y, Ohshima K, Nagashima T, Serzizawa M, Tanabe T, Saito J, Usui T, Watanabe Y, Naruoka A, Ohnami S, Ohnami S, Mochizuki T, Kushuhara M, Yamaguchi K (2016) Next generation sequencing approach for detecting 491 fusion genes from human cancer. Biomed Res 37: 51-62.

SUPPLEMENTARY METHODS

Cell Culture

All cells were grown in 4.5 g/L glucose DMEM containing 10% (v/v) Foetal Calf Serum and 1% (v/v) penicillin-streptomycin at 37°C and 5% CO_2. For Figures S2-4, NIH3T3 cells were transfected with BRAF-GFP or GFP vectors using methods described in the main text. For Figure S5, immortalised MEFs were transfected with pEGFP-C1 using 1 μg/μl polyethyleneimine (PEI; Sigma 408727) at a PEI:DNA ration of 8:1. PEI and DNA were incubated in Opti-MEM (Thermofisher, 31985062) for 10 min at room temperature and then added to cells at 60% confluency. Cells were incubated at 37°C and 5% CO_2 for 16-20 hours and then media was changed to complete culture media for a further 24 hours. Mock-transfected cells were processed in the same way except for the omission of the pEGFP-C1 plasmid. For Figure S6, cells were infected with BRAF-GFP adenoviruses as described in the main text. 48 hours after infection they were treated with 30 ng/ml PDGF-BB (Sigma, P3201) for the times indicated.

Immunofluorescence analysis

Cells were washed in PBS and then fixed in 4% paraformaldehyde (PFA)/PBS for 15 min. The PFA/PBS was removed and the cells were washed in PBS followed by permeabilisation in 0.4% Triton-X/PBS for 10 min. After washing in PBS, cells were blocked in 5%BSA/PBS for 30 min and then primary antibodies diluted in blocking solution were applied for 1 hour at room temperature. Antibodies used were: a 1:200 dilution of a mouse monoclonal BRAF antibody (Santa Cruz, SC-5284), a 1:200 dilution of a rabbit monoclonal PP-MEK antibody (Cell Signaling 9154, clone 41G9) or a 1:200 dilution of a mouse monoclonal PP-ERK antibody (clone MAPK-YT, Sigma M9692). After washing in PBS, respective secondary antibodies were applied at in blocking solution for 30 min at room temperature, using either an AlexaFluor® 546-conjugated goat anti-mouse IgG (H+L) (1:200, Thermo Fisher, A-11003) or AlexaFluor® 568-conjugated goat anti-rabbit IgG (Fab) antibody (1:200, Thermo Fisher, A11019). Cells were washed in PBS and then incubated in 1 mg/ml DAPI for 5 min, washed and mounted using ProLong™ Glass Antifade Mountant Thermo Fisher,

P36982) before microscopy. Epifluorescence microscopy was performed using an inverted Nikon TE300 microscope with Hamamatsu ORCA-ER digital camera and X-cite 120 fluorescence illumination system controlled by Improvision's Openlab software. Confocal imaging was performed using an Olympus FV1000 confocal laser scanning system on an inverted IX81 motorized microscope equipped with UPlanFLN 40x/1.3NA objective (Olympus), and the obtained images were deconvoluted using Huygens Essential software (Scientific Volume Imaging). For Figure S3, the FRAP analysis was performed as described in the Materials and Methods section of the main text. For Figure S4, the quantitation of subcellular distribution was determined as described in the Materials and Methods section of the main text. For Figure S6, HCM analysis was performed in the Materials and Methods section of the main text.

Location	Genes
Cytoplasm (24)	AKAP9 DYNC1I2 KLHL7 MZT1 RBMS3 TRIM24 EPS15 CUX1 ARMC10 SND1 NUP214 AGK ZKSCAN1 CCDC6 CCDC91 GHR TANK ETFA GTF2I MYRIP STK35 STRN3 ZC3HAV1 AGAP3
Intracellular organelle (26)	AKAP9 DYNC1I2 KLHL7 MZT1 RBMS3 TRIM24 EPS15 NUB1 CUX1 ARMC10 SND1 NUP214 AGK RAD18 CCDC6 CCDC91 GHR ETFA GTF2I MYRIP STK35 STRN3 ZC3HAV1 AGAP3
Organelle (26)	AKAP9 DYNC1I2 KLHL7 MZT1 RBMS3 TRIM24 EPS15 NUB1 CUX1 ARMC10 SND1 NUP214 JHDM1D AGK RAD18 CCDC6 ZKSCAN1 CCDC91 GHR ETFA GTF2I MYRIP STK35 STRN3 ZC3HAV1 AGAP3
Intracellular non-membrane bounded organelle (13)	JHDM1D AKAP9 DYNC1I2 KLHL7 MZT1 RAD18 TRIM24 CCDC6 CCDC91 CUYX1 GTF2I MYRIP STK35
Non-membrane bounded organelle (13)	JHDM1D AKAP9 DYNC1I2 KLHL7 MZT1 RAD18 TRIM24 CCDC6 CCDC91 CUX1 GTF2I MYRIP STK35
Microtubule organizing centre part (2)	AKAP9 MZT1
Pigment granule (2)	SND1 MYRIP
Melanosome (2)	SND1 MYRIP
Neuronal cell body (3)	GTF2I STRN3 GHR
Intracellular part (27)	AKAP9 DYNC1I2 KLHL7 MZT1 RBMS3 TRIM24 EPS15 NUB1 CUX1 ARMC10 SND1 NUP214 JHDM1D AGK RAD18 CCDC6 ZKSCAN1 CCDC91 GHR TANK ETFA GRF2I MYRIP STK35 STRN3 ZC3HAV1 AGAP3
Intracellular membrane-bounded organelle (23)	AKAP9 KLHL7 RBMS3 TRIM24 EPS15 NUB1 CUX1 ARMC10 SND1 NUP214 JHDM1D AGK RAD18 ZKSCAN1 CCDC91 GHR ETFA GTF2I MYTRIP STK35 STRN3 ZC3HAV1 AGAP3
Cell body (3)	GRF2I STRN3 GHR
Membrane bounded (23)	AKAP9 KLHL7 RBMS3 TRIM24 EPS15 NUB1 CUX1 ARMC10 SND1 NUP214 JHDM1D AGK RAD18 ZKSCAN1 CCDC91 GHR ETFA GRF2I MYRIP STK35 STRN3 ZC3HAV1 AGAP3
Cytoplasmic part (18)	AKAP9 DYNC1I2 MZT1 TRIM24 EPS15 CUX1 snd1 ARMC10 NUP214 AGK ZKSCAN1 CCDC91 TANK GHR ETFA MYRIP STRN3 ZC3HAV1
Intracellular (27)	AKAP9 DYNC1I2 KLHL7 MZT1 RBMS3 TRIM24 EPS15 NUB214 JHDM1D AGK RAD18 CCDC6 ZJSCAB1 CCDC91 GHR TANK ETFA GTF2I MYRP STK35 STRN3 ZC3GAV1 AGAO3
Centrosome (3)	AKAP9 DYNC1I2 MZT1
Nucleus (16)	KLHL7 TRIM24 NUB1 CUX1 SND1 NUP214 JHDM1D RAD18 ZKSCAN1 CCDC91 GHR GRF2I ZC3HAV1 STRN3 STK35 AGAP3
Nuclear lumen (9)	JHDM1D KLHL7 TRIM24 CCDC91 CUX1 GTF2I NUP214 STK35 STRN3
Intracellular organelle part (17)	AKAP9 DYNC1I2 KLHL7 MZT1 TRIM24 EPS15 CUX1 ARMC10 NUP214 JHDM1D AGK RAD18 CCDC91 ETFA GTF2I STK35 STRN3
Mitochondrion (6)	AGK ARMC10 SND1 ZKSCAN1 GHR ETFA
Organelle part (17)	AKAP9 DYNC1I2 KLHL7 MZT1 TRIM24 EPS15 CUX1 ARMC10 NUP214 JHDM1D AGK RAD18 CCDC91 FTFA GTF2I STK35 STRN3
Nucleolus (6)	JHDM1D KLHL7 GTF2I CCDC91 STK35 CUX1
Intracellular organelle lumen (10)	JHDM1D KLHL7 TRIM 24 CCDC91 ETFA CUX1 GTF2I NUP214 STK35 STRN3
Golgi apparatus (5)	AKAP9 CCDC91 STRN3 ZC3HAV1 CUX1
Microtubule organizing centre (3)	AKAP9 DYNC1I2 MZT1
Organelle lumen (10)	JHDM1D KLHL7 TRIM24 CCDC91 ETFA CUX1 GTF2I NUP214 STK35 STRN3
Cilium (2)	MYRIP EPS15
Nuclear part (9)	JHDM1D KLHL7 TRIM24 CCDC91 CUX1 GTF2I NUP214 STK35 STRN3
Vesicle (4)	DYNC1I2 SND1 MYRIP EPS15
Chromatin (2)	RAD18 TRIM24

Table S1. Cellular component ontogeny analysis of BRAF translocation partners detected in human cancers. The software tool WebGestalt (www.webgestalt.org/option.php) was used for gene ontology enrichment analysis to analyse genes that comprise translocation partners of *BRAF* in various human cancers. Translocation partner genes were identified from COSMIC (http://cancer.sanger.ac.uk/cosmic/fusion) or publications: Latysheva and Babu (2016), Ross et al (2016) or Urakami et al (2016). The cellular component describes one aspect of gene ontology, depending on where the gene products are active.

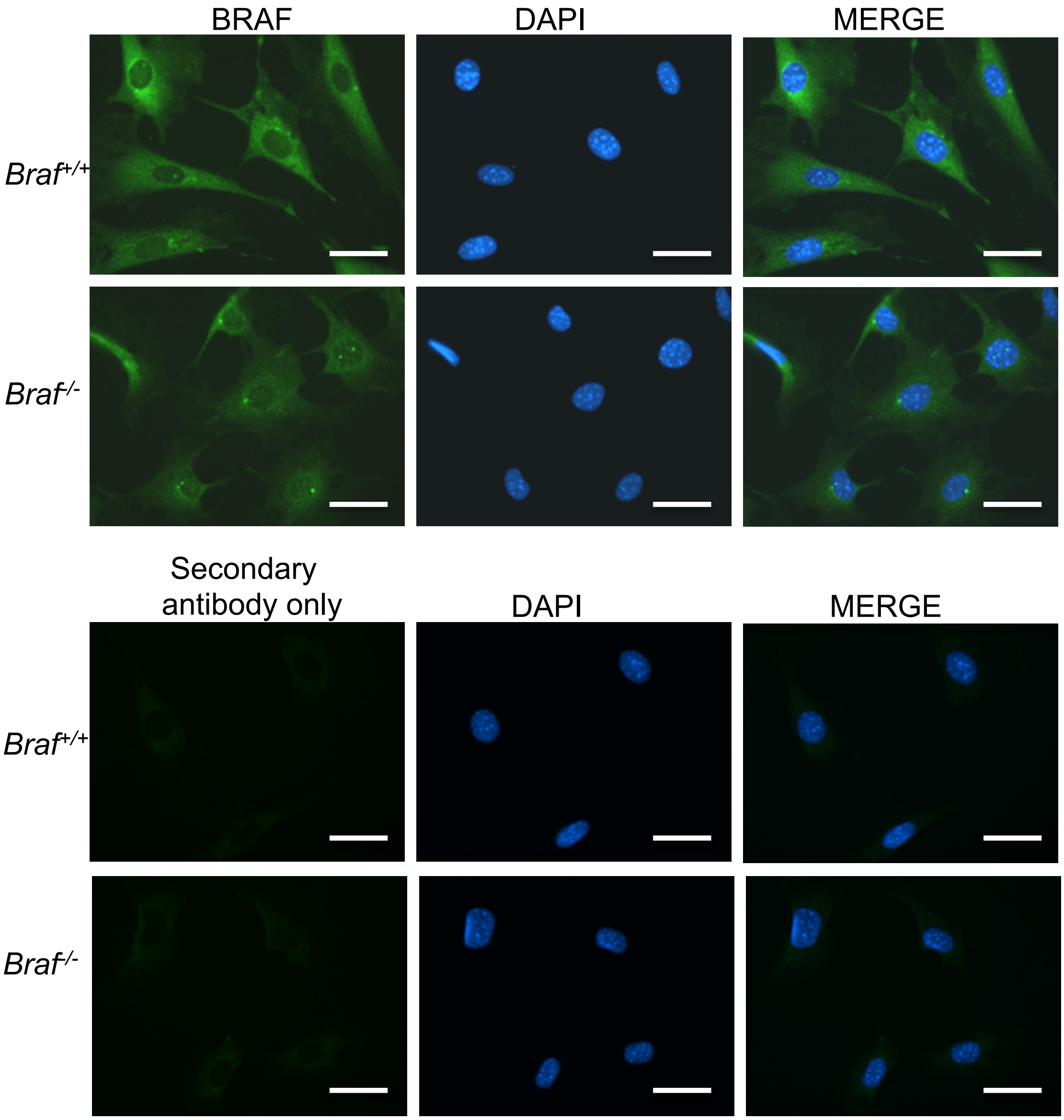

Figure S1. Immunofluorescence analysis of endogenous BRAF. *Braf*$^{+/+}$ and *Braf*$^{-/-}$ immortalised MEFs were immunostained with a BRAF antibody or with the secondary antibody alone. Cells were counterstained with DAPI, analysed by epifluorescence microscopy and the images merged. We have previously shown that this antibody detects a single protein species at ~90KDa in *Braf*$^{+/+}$ MEFs by western blot analysis that is absent from *Braf*$^{-/-}$ MEFs (Noble et al. 2008). A similar staining pattern is observed in the *Braf*$^{+/+}$ and *Braf*$^{-/-}$ MEFs indicating that the staining with this antibody is not entirely specific for BRAF. Scale bars = 50 μm.

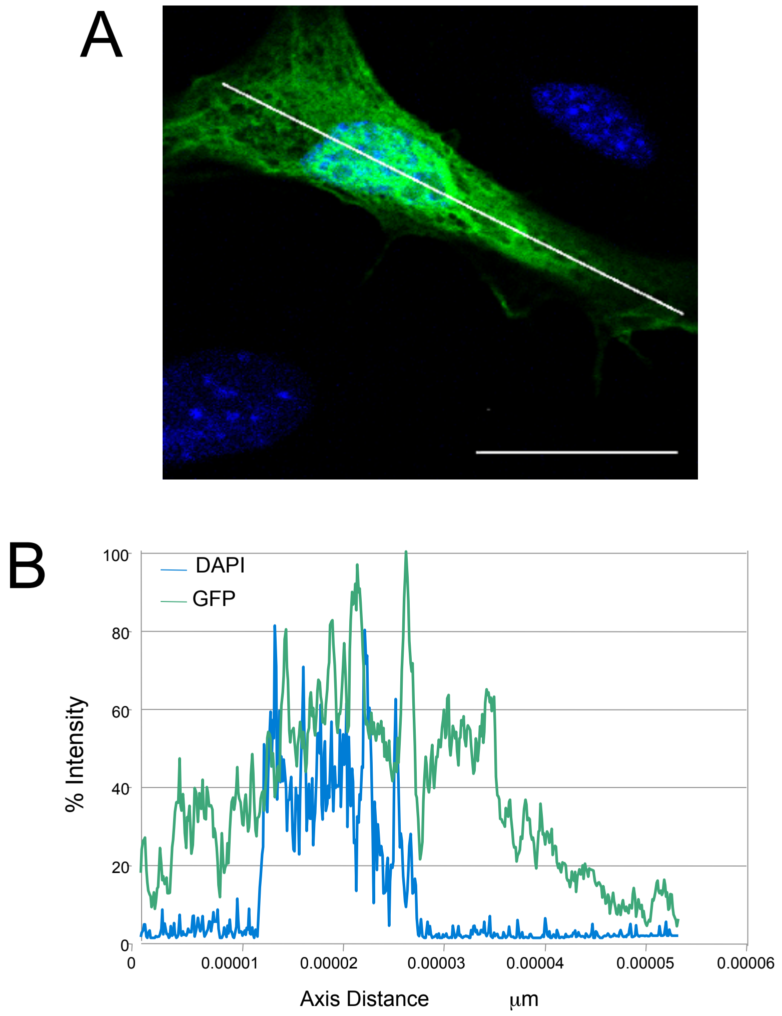

Figure S2. Confocal analysis of GFP-ΔBRAF. Z section of a nuclear-stained cell. NIH3T3 cells were transfected with GFP-ΔBRAF and a cell with predominantly nuclear GFP staining was subjected to confocal analysis. A Z series of 0.3 μm stacks was performed through the cell. The image in A shows a representative Z section through the middle of the nucleus. The graph in B shows the % intensity of DAPI and GFP fluorescence along the line indicated in A.

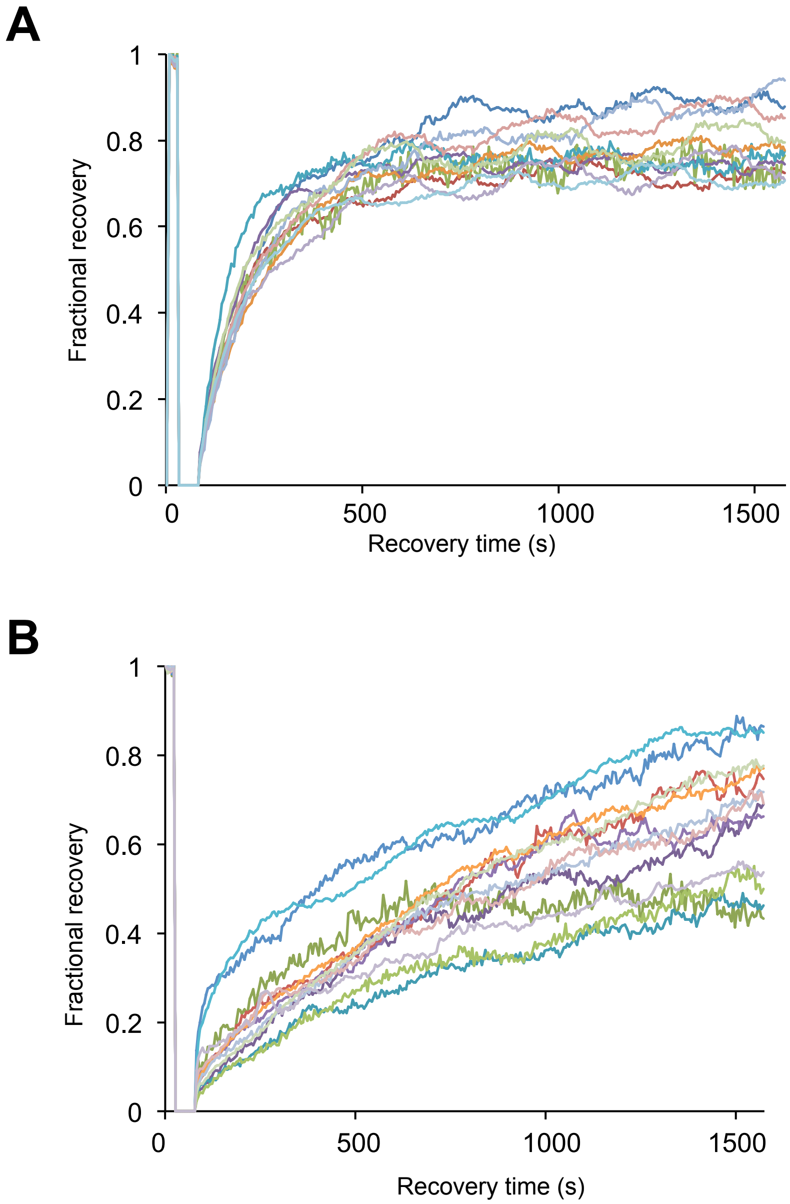

Figure S3. Individual FRAP analysis of GFP and GFP-ΔBRAF. NIH3T3 cells were transfected with a vector expressing GFP-ΔBRAF (A) or a vector expressing monomeric GFP (B) and the cells were subjected to FRAP analysis as described in the Materials and Methods section of the main text. The recovery of the bleached nucleus was recorded by collecting 300 single optical sections with a time interval of 5s for 13 cells (GFP-ΔBRAF) or 11 cells (GFP). Recovery profiles for individual cells for GFP (A) or GFP-ΔBRAF (B) are shown.

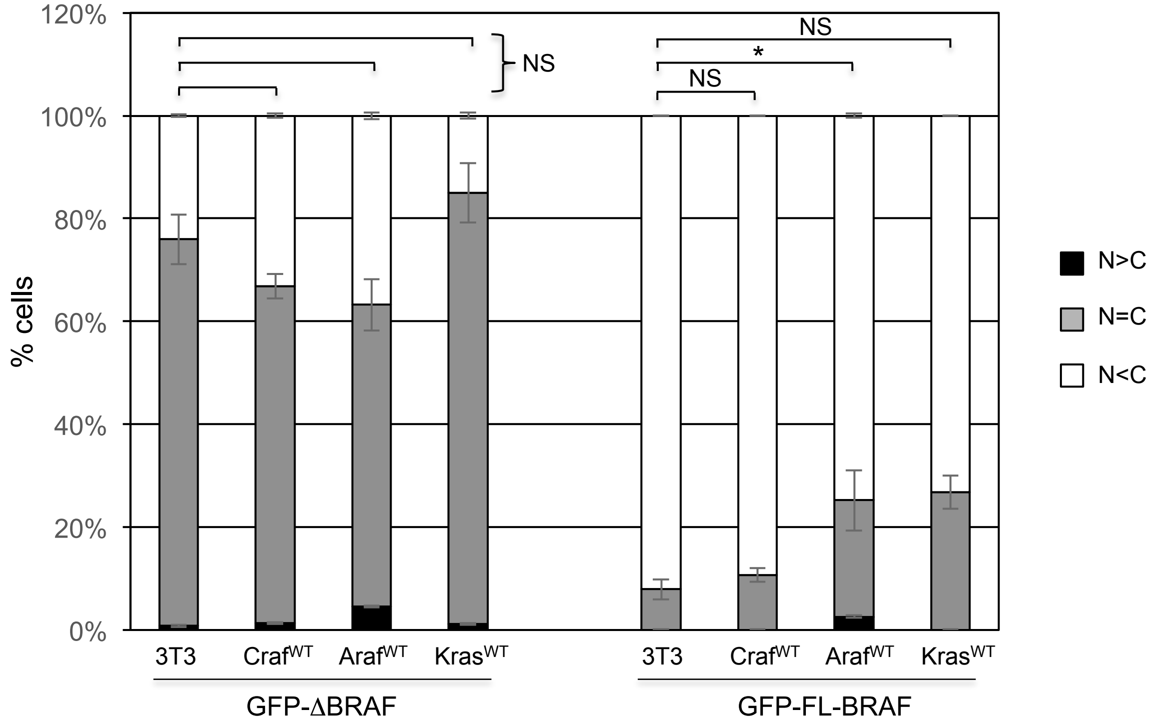

Figure S4. Comparison of GFP-BRAF distributions in different cell types. Vectors expressing GFP-ΔBRAF or GFP-FL-^{WT}BRAF were transfected into four different cell types: NIH3T3 cells or wild-type MEFs derived from different mouse crosses (Craf^{WT}, Araf^{WT}, or Kras^{WT}). Following fluorescence microscopy, GFP fluorescence was categorized by the user as mostly nuclear (N>C), equally distributed (N=C) or mostly cytoplasmic (N<C). Over 200 cells were visualized for each transfection. Bar chart indicates mean (n=3) ± SEM. Data presented is the same as that shown in Fig. 1B (NIH3T3), Fig. 5A (Craf^{WT}), Fig. 5B (Araf^{WT}) or Fig. 5C (Kras^{WT}). The data show no significant difference (NS) for the majority of cell lines except for GFP-FL-^{WT}BRAF which showed significantly higher proportion of N>C and N=C cells in Araf^{WT} MEFs compared to NIH3T3 cells.

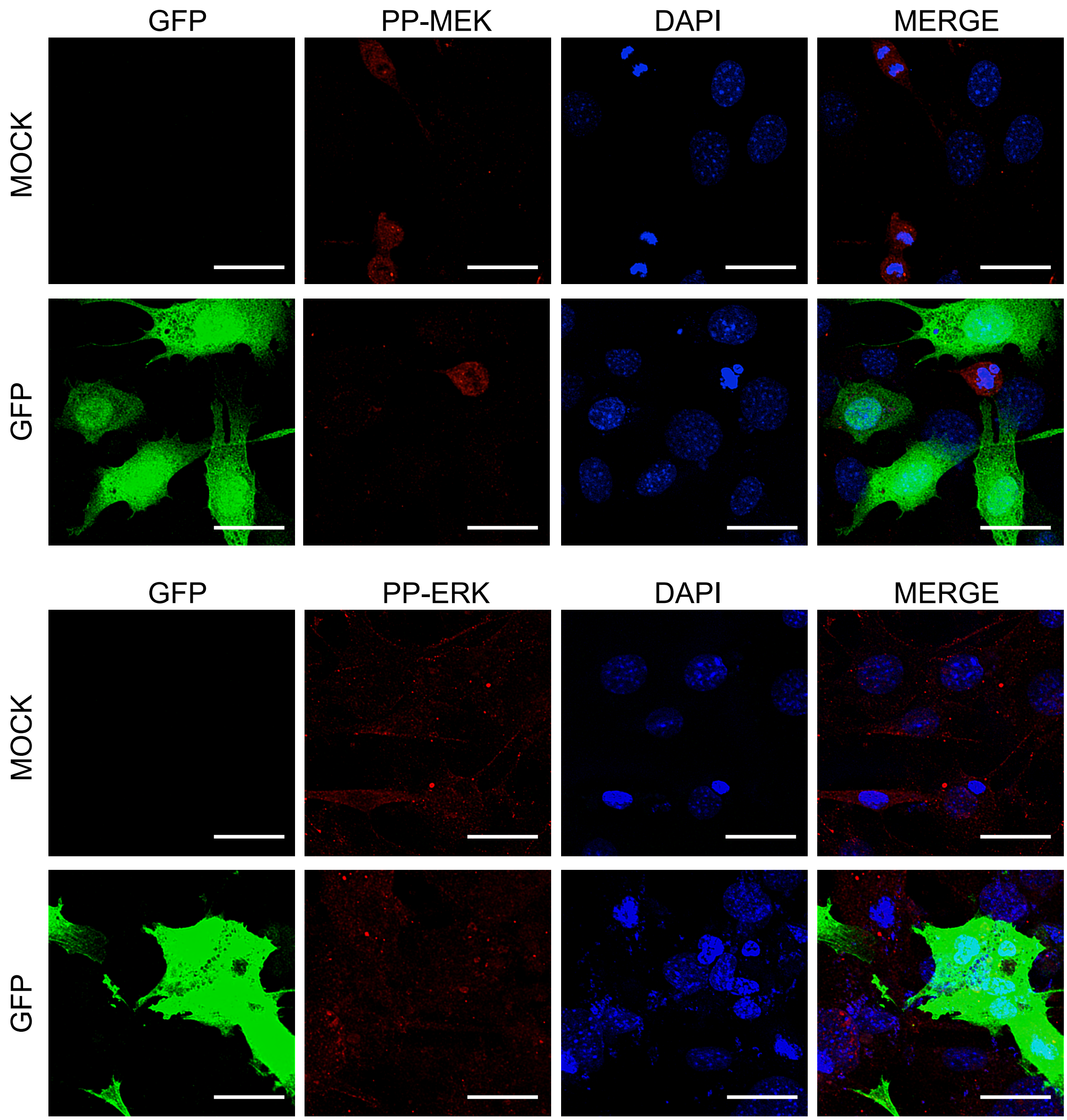

Figure S5. Immunostaining for phosphorylated MEK and ERK in control transfected cells. Immortalised fibroblasts were either mock transfected or transfected with the pEGFP-C1 vector. 48 hours after transfection cells were processed for immunostaining with PP-MEK and PP-ERK antibodies and counterstained with DAPI. Cells were analysed with a confocal microscope and representative areas photographed. GFP, PP-MEK, DAPI and merged images are shown in the top panels and GFP, PP-ERK, DAPI and merged images in the bottom panels. The data in the top panels show no PP-MEK immunostaining in mock/GFP-transfected cells except for low levels of cytoplasmic staining in mitotic cells. The data in the bottom panels show similar levels of very low intensity non-nuclear PP-ERK staining in mock/GFP-transfected cells. Scale bars = 20μm.

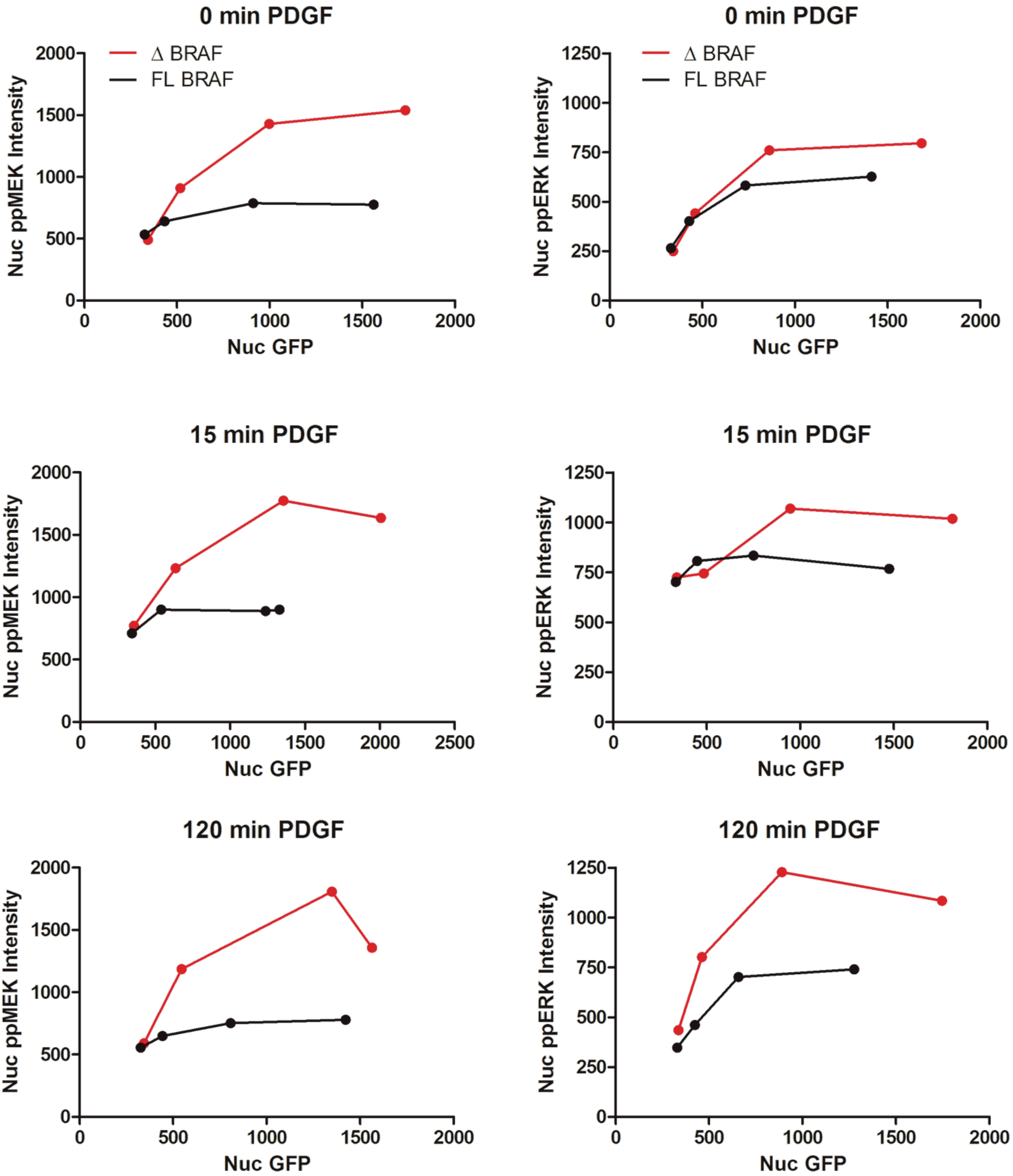

Figure S6. Phosphorylated MEK and ERK localisation following PDGF stimulation using High Content Microscopy. NIH3T3 cells were infected with adenoviruses expressing either ΔBRAF-GFP or GFP-FL-^{WT}BRAF. 48 hours after infection, cells maintained in 10% FCS were treated with PDGF for the times indicated. Cells were immunostained for PP-MEK or PP-ERK, counterstained with DAPI and subjected to HCM analysis. Graphs represent population average normalised values for nuclear PP-MEK or PP-ERK plotted against values for nuclear GFP. Data shown are population averages from three experiments performed in duplicate and are in AFU.

Original Western Blots

Figure 1C

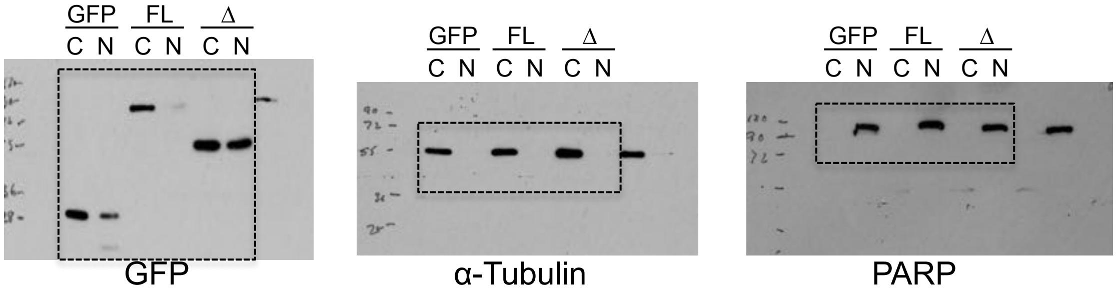

Figure 1D

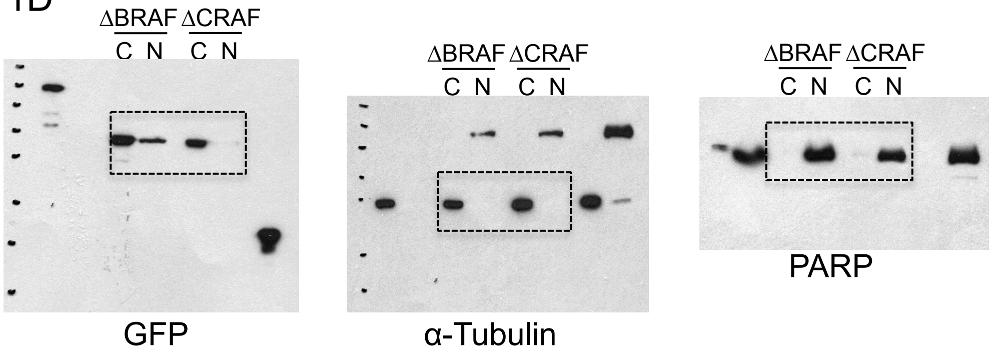

Figure 1E

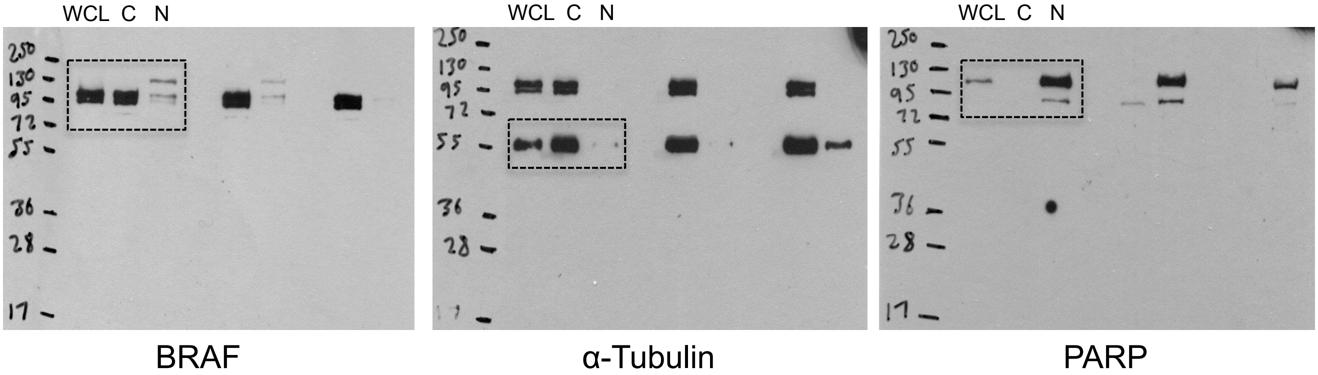

Figure 2C

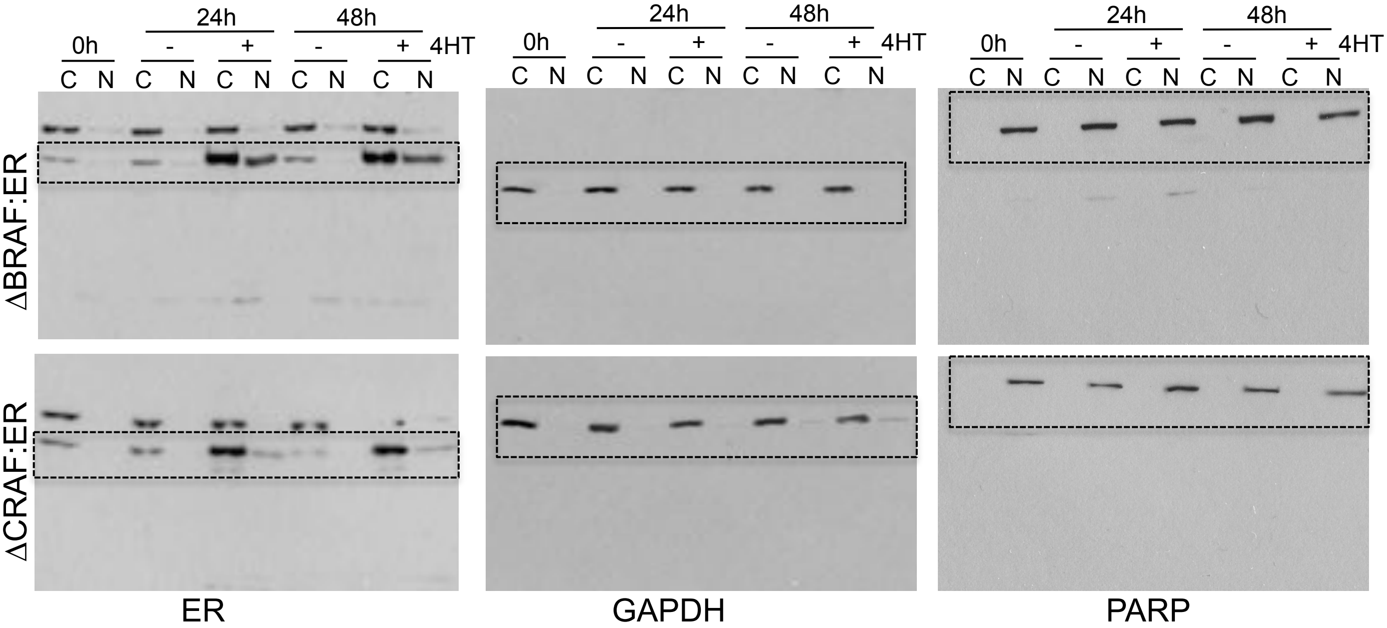

Figure 3B

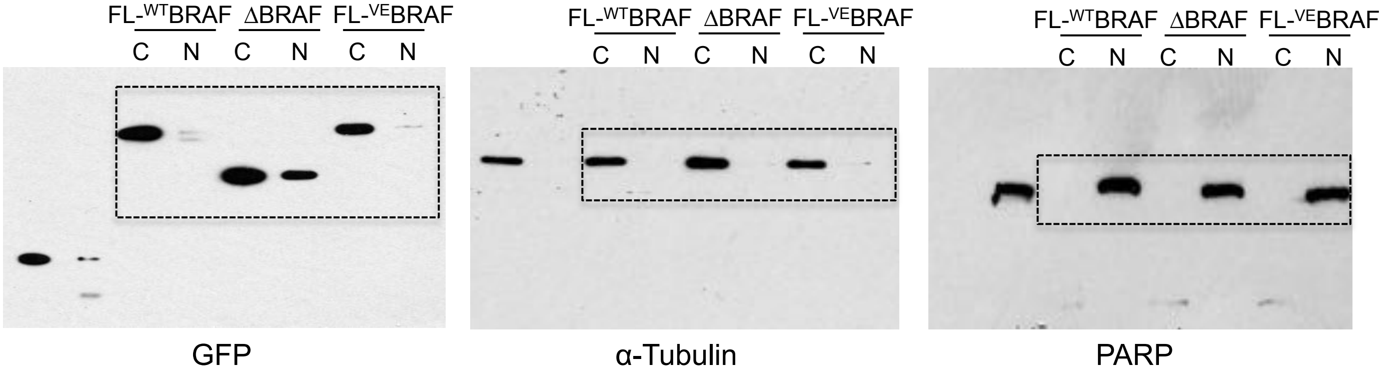

Figure 3C

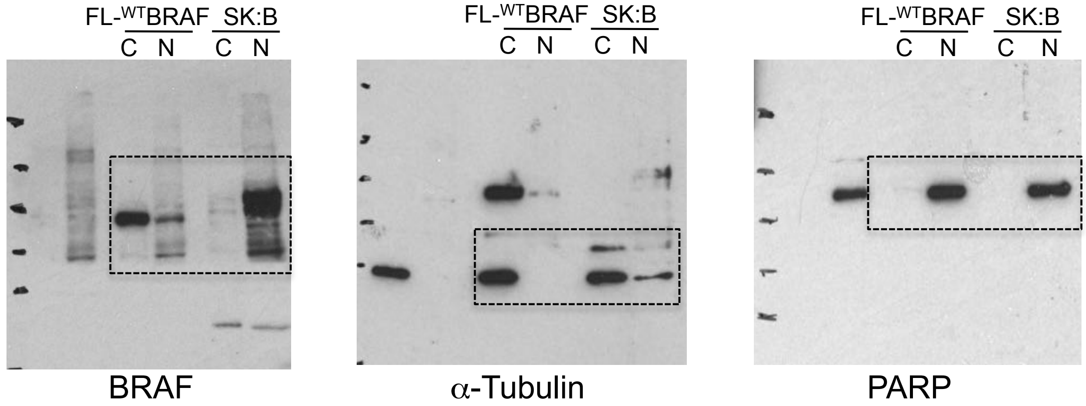

Figure 4C

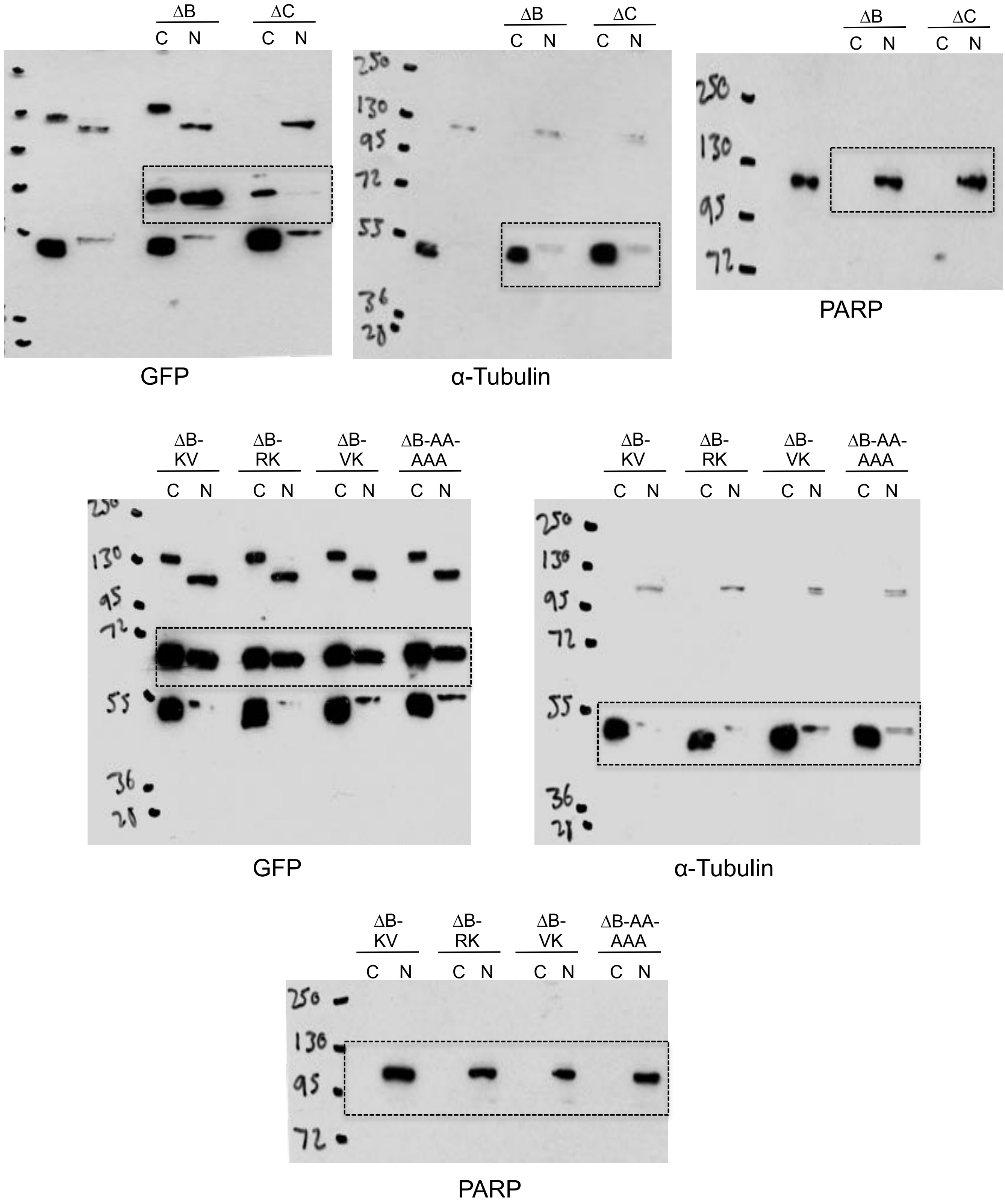
ΔB ΔC
C N C N
GFP
250 130 95 72 55 36 28
α-Tubulin
PARP
ΔB-KV ΔB-RK ΔB-VK ΔB-AA-AAA
C N C N C N C N
GFP
α-Tubulin
PARP

Figure 5E

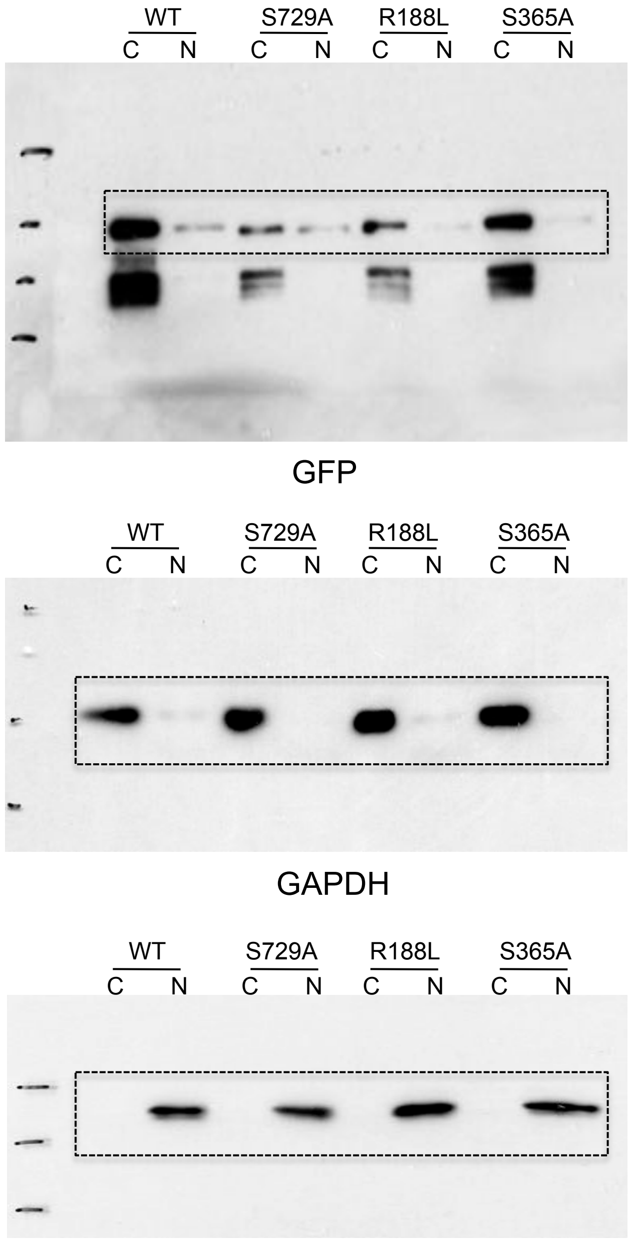

Figure 6C

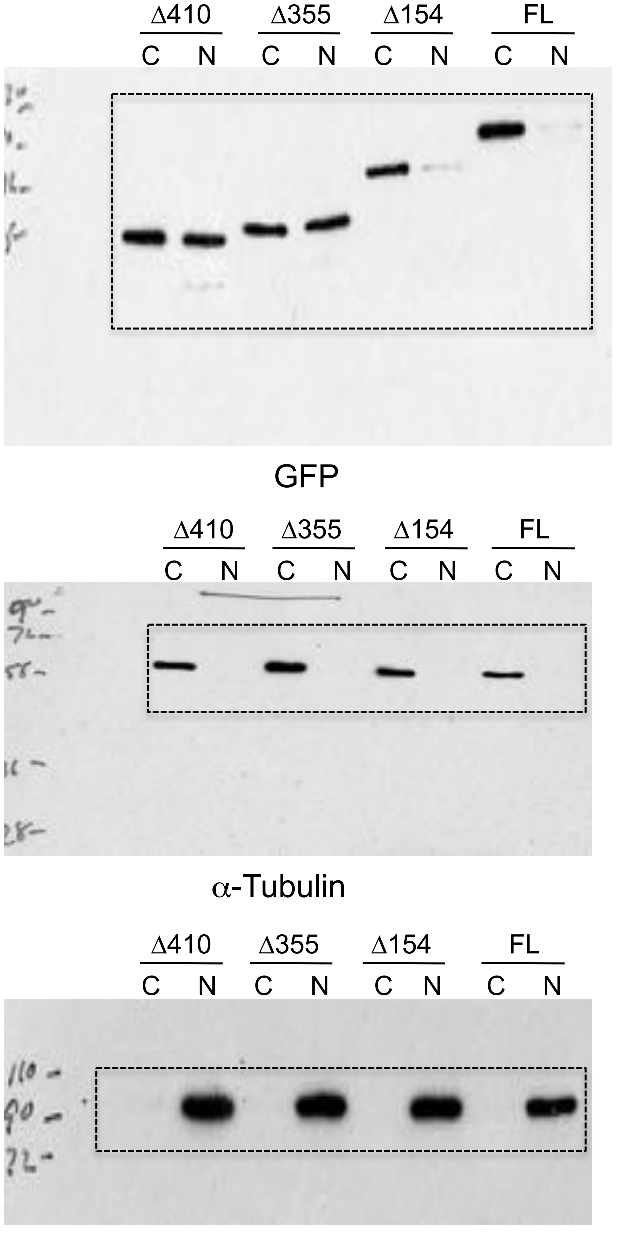

Figure 7B

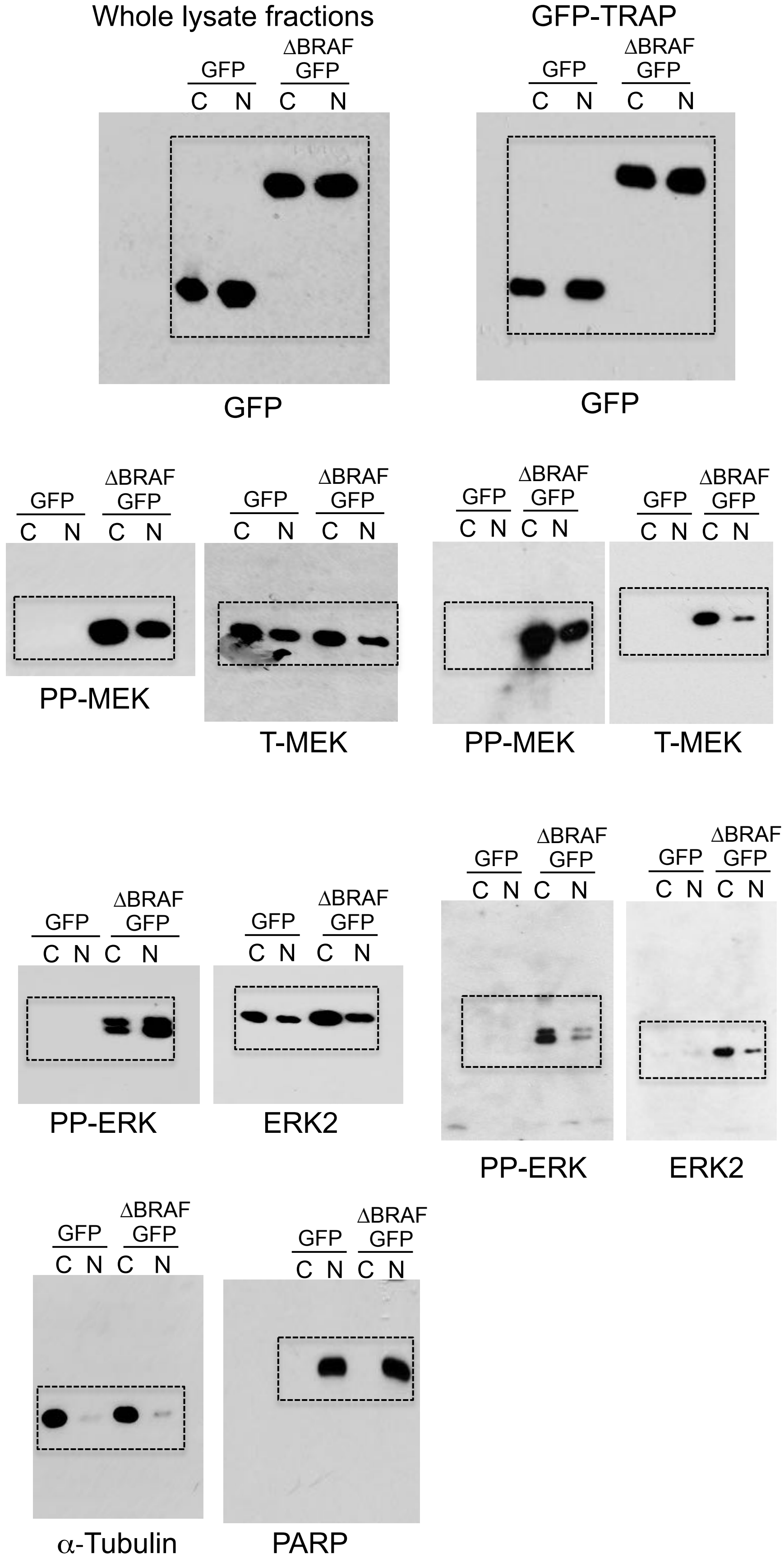

Figure 7A

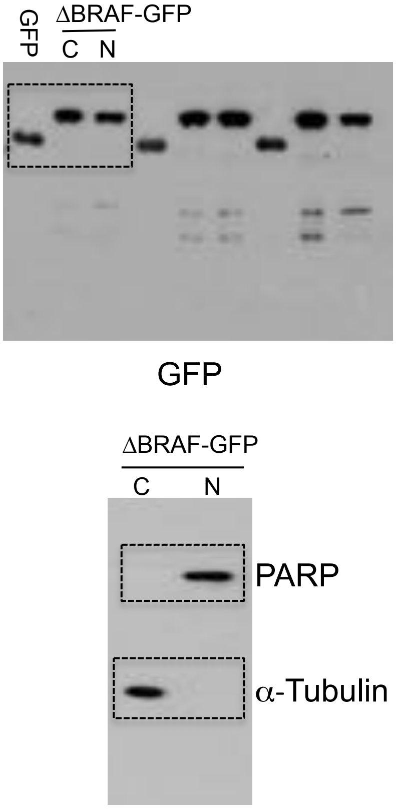

Figure 7C

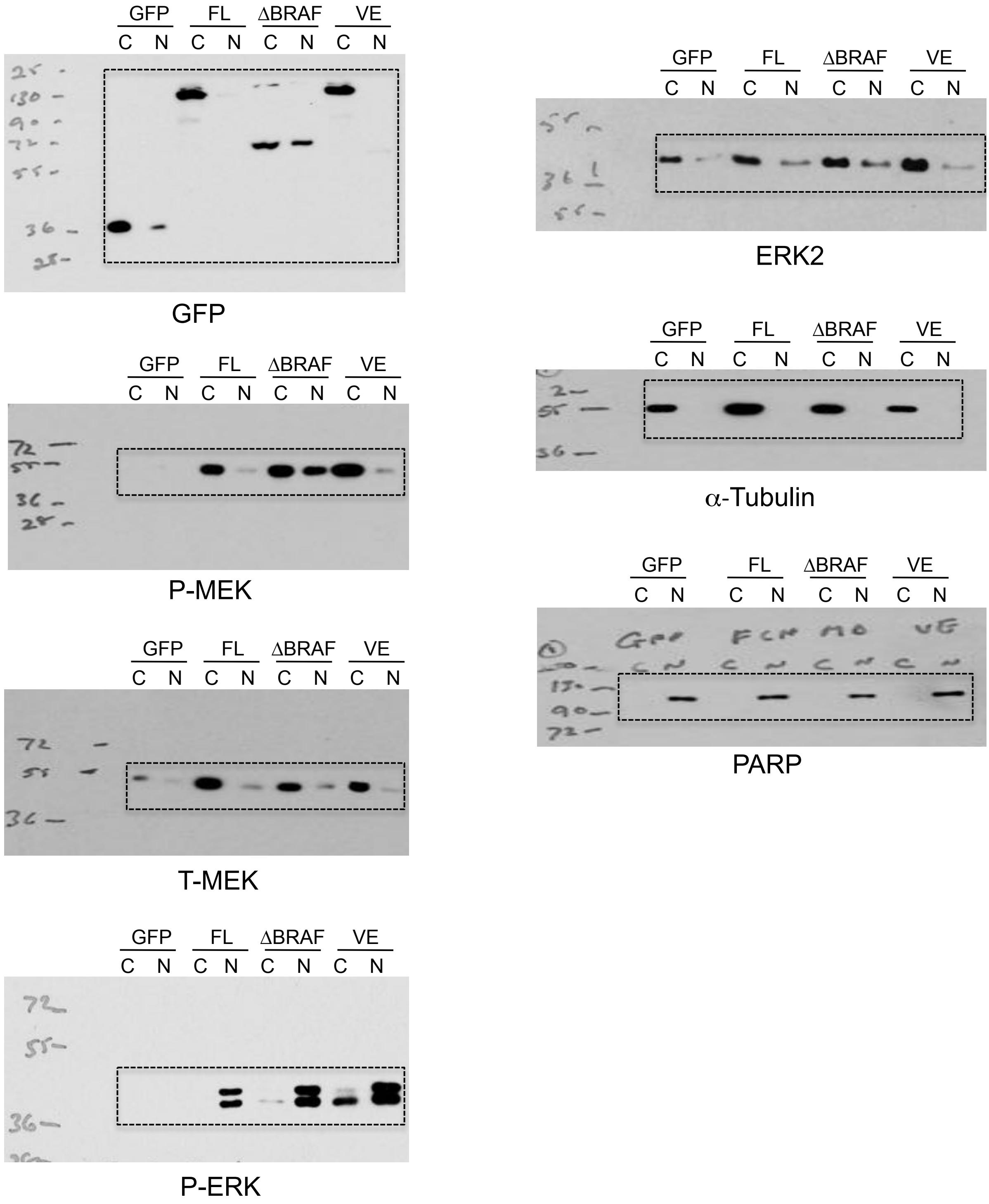
GFP FL ΔBRAF VE
C N C N C N C N
GFP
P-MEK
T-MEK
P-ERK
ERK2
α-Tubulin
PARP
